# Understanding Polysiloxane Polymer to Amorphous SiOC Conversion During Pyrolysis Through ReaxFF Simulation

**DOI:** 10.3390/ma18071412

**Published:** 2025-03-22

**Authors:** Kathy Lu, Harrison Chaney

**Affiliations:** 1 Department of Mechanical and Materials Engineering, EEC Building 257, University of Alabama at Birmingham, Birmingham, AL 35294, USA; 2 Department of Materials Science and Engineering, 260 Holden Hall, Virginia Polytechnic Institute and State University, Blacksburg, VA 24061, USA; hmchaney@vt.edu

**Keywords:** polymer to ceramic conversion, atomic evolution, molecular structure, ReaxFF simulation, cluster size, composition separation

## Abstract

A significant challenge during the polymer-to-ceramic pyrolysis conversion is to understand the polymer-to-ceramic atomic evolution and correlate the composition changes with the precursor molecular structures, pyrolysis conditions, and resulting ceramic characteristics. In this study, a Reactive Force Field (ReaxFF) simulation approach has been used to simulate silicon oxycarbide (SiOC) ceramic formation from four different polysiloxane precursors. For the first time, we show atomically that pyrolysis time and temperature proportionally impact the new Si-O rich and C rich cluster sizes as well as the composition separation of Si-O from C. Polymer side groups have a more complex effect on the Si-O and C cluster separation and growth, with ethyl group leading to the most Si-O cluster separation and phenyl group leading to the most C cluster separation. We also demonstrate never-before correlations of gas release with polymer molecular structures and functional groups. CH_4_, C_2_H_6_, C_2_H_4_, and H_2_ are preferentially released from the pyrolyzing systems. The sequence is determined by the polymer molecular structures. This work is the first to atomically illustrate the innate correlations between the polymer precursors and pyrolyzed ceramics.

## 1. Introduction

With the continuous demand for novel high temperature materials, polymer-derived high temperature ceramics are attracting significant attention. They offer a wide range of synthesis flexibilities, shape factors, and never-before microstructures and properties through tailorable polymer precursor chemistry and compositions [1,2,3,4] as well as processing conditions [1,5,6,7]. This is especially the case for harsh environmental conditions as the resulting microstructures, cluster sizes, and phase distributions can be tuned by using different precursors and pyrolysis conditions.

One specific family of polymer-derived ceramics is SiOC. Such materials are made from polysiloxane (PSO) polymers. During pyrolysis in vacuum, inert, or reducing atmospheres, continuous compositional and microstructural evolution occurs, which initiates around 400–600 °C and is accompanied by polymer chain break-up, evaporative species release, and radical species reorganization and re-bonding [8]. At 900–1000 °C, SiOC, SiO_2_, and free carbon phases form, resulting in nanoscale composition separation [9]. Due to the drastic atomic structure and compositional changes from polymer to SiOC ceramics, there has not been systematic examination of the early stage of the polymer to ceramic conversion process. The complex atomic structures of SiO_x_C_y_ (0 ≤ x, y ≤ 4) and different carbon phases as well as the compositional similarity (in atomic numbers or mixtures of various similar amorphous phases) lead to a severe lack of understanding of the continuously evolving atomic structures. As a result, the genesis and mechanisms of new phase formation as well as the specific path of atomic evolution of the resulting ceramics remain to be understood.

Since the conversion from polymer to amorphous- and nano-ceramics is experimentally complex and intractable, obtaining atomic-level understanding and tuning demands a more modeling-based approach, especially considering the myriad of polymer precursors, pyrolysis conditions, and the complexity of the pyrolyzed microstructures. The specific variables to consider include starting precursors with different molecular structures, compositions, and chemical bonds, as well as pyrolysis conditions that can be widely varied, such as pyrolysis temperature and time [10]. At high temperatures, the evaporative release of organic groups and various redistribution reactions between Si-O, Si-C, and Si-H bonds result in the formation of low-molecular-weight oligomers. Subsequently, the cleavage and volatilization of the organic groups in the form of hydrocarbons and H_2_ take place [11]. The mechanisms are complex, multiple events often occur simultaneously. Until now, experimental studies have been sporadic. One study [12] claimed that silicon atoms are removed as volatile silanes and carbon atoms are lost as CH_4_. Other studies [13,14,15] either used a reactive atmosphere to accelerate certain reactions or just considered the kinetics of gas release, which did not shed much light on the intrinsic behaviors of polymer conversion to ceramics.

ReaxFF, a molecular dynamics method, can simulate polymer precursor to ceramic conversion at the atomic level [16,17,18]. ReaxFF uses bond-order dependent force fields to model bond breaking and formation during polymer-to-ceramic transformation. It can simulate thermal decomposition and evolution of volatiles as well as structural reorganization at high temperatures. The first step in the simulation involves building molecular models of polymer precursors, such as Si-O-C-H based PSO. ReaxFF allows tracking the atomic rearrangements and chemical transformations by monitoring chemical composition, elemental ratios, formation of SiC_x_C_y_ network structures, growth of ceramic domains, Radial Distribution Function (RDF) [19,20], gas phase analysis, etc. It originated from simulations of the initial oxidation process of a SiC surface exposed to O_2_ and H_2_O molecules [21,22,23,24]. The desirable feature lies in an atomically detailed reactive molecular dynamics method that can model the breaking and formation of bonds. The approach describes Si–Si, Si–O, Si–H, Si–C–O, and Si–O–H bond interactions; it can also simulate C–C bond formation [21,22,23,24,25]. A ReaxFF interatomic potential has been proposed to account for all interactions [21,22,23,24,25]: *E*_system_ = *E*_bond_ + *E*_over-coordination_ + *E*_under coordination_ + *E*_valence angle_ + *E*_penalty_ + *E*_torsion_ + *E*_conjugated energy_ + *E*_vdWaals_ + *E*_Coulomb_ (*E*_system_: system energy; *E*_bond:_ bond energy; *E*_over-coordination_: over coordination energy; *E*_under coordination_: under coordination energy; *E*_valence angle_: valence angle energy; *E*_penalty_: penalty energy; *E*_torsion_: torsion energy; *E*_conjugated energy_: conjugation energy; *E*_vdWaals_: van der Waals energy; *E*_Coulomb_: Coulomb energy). The potential energy functions for each of these energy terms are developed individually. This force field simulates Si–C–O bond tendencies and allows the evaluation of polymer precursor effects on compositions and phase distributions of the resulting SiOC systems. Furthermore, the ReaxFF method is fast and allows larger-scale simulations of chemical interactions [21,23]. Because of these intentional and detailed attributes, ReaxFF provides an incredible opportunity to tackle the challenges of correlating polymer precursors with the pyrolyzed ceramics starting at the atomic level.

Our earlier work [26], using the ReaxFF approach, showed that after the polymer precursor conversion to ceramics, SiO_2_ regions need to be of a large enough size to experience O reduction and C diffusion. The work also unified the carbothermal reaction process between SiO_2_ clusters and C domains for SiC formation. C cluster growth is inversely related to removal of O. The C-rich precursor forms C-C bonds through Si-O, Si-C, and C-H bond losses while less C-rich polymers have no significant C-C bond formation during C-H bond loss [27]. There is also the presence of a small fraction of Si-Si bonds, which has never been addressed before. The end structures have vastly different Si-O rich and C-rich domain sizes. Our work also showed that the perceived SiOC phase separation is non-existent. Instead, SiO_2_ and free carbon clusters form first and then carbothermal reduction sets in to induce SiOC formation [28].

In this study, polymer precursor conversion to SiOC from 1800 to 2100 K was simulated using ReaxFF. To understand the precursor molecular structure effects, four polymer precursors were studied: polydimethylsiloxane (PDMS), poly(vinylmethylsiloxane) (PVMS), polydiethylsiloxane (PDES), and poly(methylphenylsiloxane) (PMPS), with side group size or C bond differences. The novelty of this study lies in its comprehensive investigation of the key parameters influencing the atomic structure evolution of SiOC during pyrolysis. Specifically, the effects of pyrolysis time, temperature, precursor molecular structure, and gas release were systematically analyzed to reveal their interdependencies and impact on SiOC formation. Unlike previous studies focusing on specific species, such as free carbon, this work provides a correlated understanding of how these variables collectively dictate the structural transformation at the atomic level, offering new insights into the controlled synthesis of SiOC materials.

## 2. Simulation Methods

**Figure 1:**
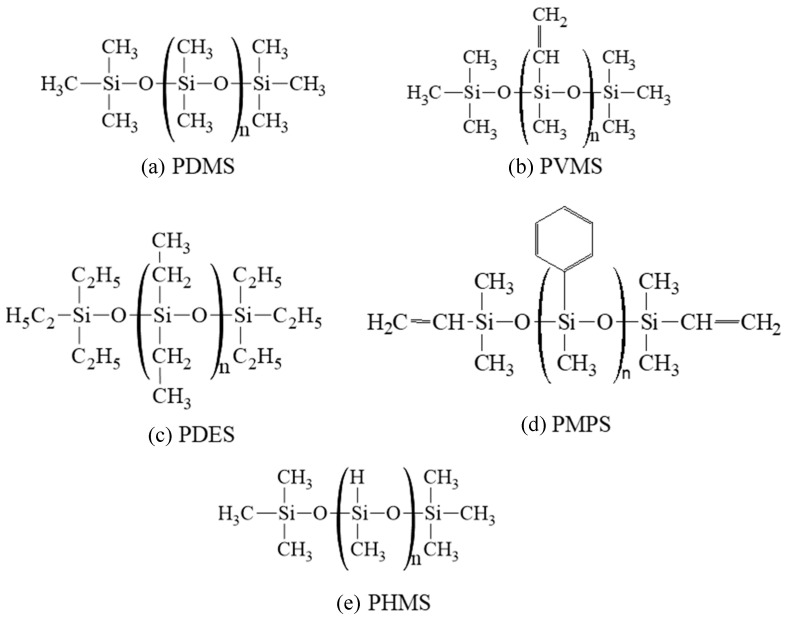
Molecular structures of the five polymer precursors used in this study: (**a**) PDMS, (**b**) PVMS, (**c**) PDES, (**d**) PMPS, and (**e**) PHMS.

The molecular structures of the precursors studied are shown in Figure 1. Pure PDMS and PDES systems were used in this study. For the PVMS and PMPS systems, 15 wt% polyhydromethylsiloxane (PHMS) was added to each system in order to adjust the C content in the pyrolyzing systems. As a result, the C contents for the repeating units in PDMS, PVMS/PHMS, PDES, and PMPS/PHMS are 32.39%, 38.54%, 47.00%, and 55.45%, respectively, in increasing order. From this point on, PVMS/PHMS and PMPS/PHMS are simply labeled as PVMS and PMPS for brevity, also because the PVMS and PMPS functional groups were adjusted to reflect the 15 wt% PHMS addition, not by creating two separate precursors. To create these polymer precursor structures for the ReaxFF simulations, we wrote simulation codes using Python to generate an idealized linear structure of Si-O first. The backbone of the initial structure consisted of 100 Si atoms and 100 O atoms. Different side groups, -H, -CH_3_, -C_2_H_5_, and -C_6_H_5_, were then distributed on the Si-O backbone according to the compositions of different polymer precursors.

Large-scale Atomic/Molecular Massively Parallel Simulator (LAMMPS) [29] was used for the ReaxFF [22,23] molecular dynamics simulation. At the start of the simulation, the linear structures shown in Figure 1 exhibited considerable strain. To alleviate this, the system’s pressure was set to 0 atm, allowing the structures to relax. Once relaxed, the polymer chain was replicated seven times, resulting in a total of eight relaxed chains. These chains were then randomly oriented while aligning their centers to form a simple cubic stacking arrangement. Following this, an additional relaxation step was conducted, enabling the polymer chains to reorganize and develop into a dense amorphous structure. To facilitate this process, the system was subjected to a constant-temperature, constant-pressure (NPT) simulation, where it was heated to 300 K under a pressure of 2 atm. The system was then run for 250,000 timesteps, with each timestep set at 0.2 fs.

The “relaxed” and “condensed” structures were further scaled up to include 96,768 atoms for PDMS, 84,352 atoms for PVMS, 102,912 atoms for PDES, and 93,438 atoms for PMPS. This was achieved by duplicating the smaller, relaxed configurations and subsequently subjecting them to an NPT simulation. Over a duration of 0.5 ns, the system was gradually heated from 300 K to 1500 K, 1800 K, and 2100 K in three separate parallel simulations. Because different gasses are generated, slow heating is needed in order to prevent the system from exploding. This is consistent with our experimental work. We mostly use 1 °C/min heating rate to prevent the sample from cracking. Throughout each heating phase, the pressure was kept at 2000 bar to prevent excessive system expansion. In actual experiments, 2000 bar (2000 MPa) is high. However, this high pressure was used so that we could understand and investigate other aspects of the pyrolysis process. The timestep between the simulation runs was 0.2 fs. Various gas species, such as CH_4_, H_3_C-CH_3_, H_2_C=CH_2_, H_2_, CO, CO_2_, H_2_O, O_2_, CH_2_O, and C_6_H_6_, were periodically detected during the simulation. Molecules with molecular weights corresponding to these species were assigned to a designated gas group in LAMMPS and subsequently removed at 5 ps intervals using a Python script. In our study, each gas has been uniquely identified. For example, CO has a molecular weight of 28.01010. C_2_H_4_ has a molecular weight of 28.05316. By differentially quantifying the molecular weight to the fifth decimal place, each gas species can be separated. The reason that the gas species need to be removed is because they were gasses, cannot be incorporated back into the solid, and would escape from the system during experiments. In simulation, we need to remove them to reflect the actual process.

After reaching their peak temperatures, the systems were maintained at those temperatures for 2 ns while keeping the pressure constant at 2000 bar. During this period, gas species continued to be removed at 5 ps intervals. Following the holding phase, the temperature was gradually reduced back to 300 K over 0.5 ns, resulting in a total simulation time of approximately 2.75 to 3 ns. Throughout the heating process, bonding data were recorded every 5 ps, and atomic positions were continuously tracked. More details on ReaxFF simulation of the PSO pyrolysis to SiOC systems can be found in other references [26,28]. Compared to these earlier efforts, this study introduced several novel insights: (1) atomic-level insights into cluster separation, (2) first-time correlations between gas release and polymer molecular structures, (3) comprehensive analysis of pyrolysis conditions, (4) a new understanding of side group influence, and (5) first in-depth quantitative knowledge of early-stage pyrolysis.

## 3. Results and Discussion

### 3.1. Pyrolysis Time Effect on Composition Separation and Domain Formation

There are two common observations for the pyrolyzing systems in this study. The first one is related to atomic species. After the precursor systems reach a specific pyrolysis temperature of 1500 K, 1800 K, or 2100 K, there are still plenty of H atoms present, indicating that the actual pyrolysis temperature is still relatively low, even though the simulation temperature has reached as high as 2100 K. From a different perspective, it also means that H atoms are difficult to remove from the SiOC systems. Even though H atoms are often ignored in experimental studies [8,30,31,32,33,34,35] due to its low atomic weight and the difficulty of quantification, hydrogen species can persist in the pyrolyzed systems until high temperatures, at least up to 1200 °C. Thus, H atoms are present throughout all the pyrolysis runs in this study. The second observation is related to the atomic structures. At the beginning of the peak holding temperature, all the pyrolyzed ceramic systems are largely homogeneous. With the pyrolysis time increase, the simulation shows that Si-O clusters start to separate from the C clusters. In general, longer pyrolysis time leads to more Si-O and C cluster separation. MD simulations, in general, can only simulate processes in the nanoseconds or a shorter time. When we simulate SiOC systems with ~1M atoms, a few nanoseconds can take several weeks. The only experimental condition that can be compared with is heating by some short pulse laser. In the actual systems, we expect a continuation of the cluster separation process with typical pyrolysis.

**Figure 2:**
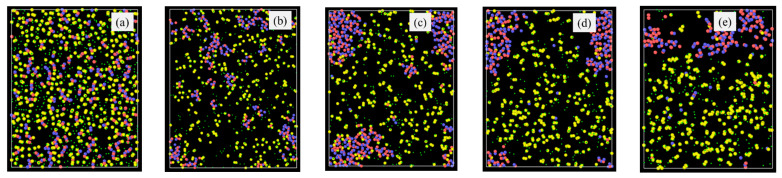
Atomic structure evolution with pyrolysis time at 2100 K simulation temperature for the PVMS system: (**a**) 0 ns, (**b**) 0.5 ns, (**c**) 1 ns, (**d**) 1.5 ns, and (**e**) 2 ns. Color scheme: Si, 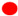; O, 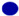; C, 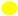; H, 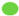.

Specifically, the atomic structural evolution for the PVMS system at 2100 K simulation temperature is shown in Figure 2 at different times. The lower pyrolysis temperatures of 1500 K and 1800 K show a similar trend but to a lesser extent as given in the supplement (Figure S1 for the 1800 K simulation results). The supplement also provides the atomic structural evolution results for PDMS (Figures S2–S4), PDES (Figures S5–S7), and PMPS (Figures S8 and S9) at different pyrolysis temperatures. In Figure 2a, the small green species are H atoms, which bond with C atoms (yellow) only. O (blue) atoms bond with Si (red) atoms with short strands resembling the Si-O linear chains in the initial polymer precursor. C atoms (yellow) bond with Si atoms, inherited from the initial polymer precursor structure, since C atoms are from the vinyl and methyl side groups. Some C species are separated from the Si species. With the simulation time increase to 0.5 ns (Figure 2b), Si-O species grow and form clusters with some remaining short Si-O strands, C-Si bonds decrease, and C atoms detach from Si atoms and form duplets or exist individually. Some C and H atoms are bonded with the C atoms from above or below the plane at display. At 1.0 ns simulation time (Figure 2c), the Si-O clusters continue to grow, with an observable amount of C atoms enclosed in the Si-O clusters. Away from the Si-O clusters, C atoms start to form duplets or triplets. Some O atoms are bonded to the C atoms, which means some gaseous species are forming. Some Si atoms are also bonded with C atoms, which means that the C side group separation is not complete. At 1.5 ns simulation time (Figure 2d), the results indicate that the Si-O clusters continue to grow in size and decrease in numbers. C duplets, triplets, and quadruplets continue to increase as a result of H removal and new C-C bond formation. Some O atoms are still bonded with C atoms, indicating continuous gaseous species formation, possibly as CO or CO_2_. Si-C bonds are very few, meaning almost complete C side group separation from the Si-O backbone. Also, the amount of C atoms in the Si-O rich clusters decreases, further indicating the C side group separation from the Si-O backbone. At 2.0 ns simulation time (Figure 2e), the simulation shows that only one large Si-O cluster is observed. In the C-rich regions, there are some C-O bonds and a few Si-C bonds. The Si-O rich cluster has very few C species. This atomic structure evolution indicates that Si-O bonds mostly separate from C atoms to form SiO_x_ clusters. Simultaneously, C atoms form amorphous C or even graphitic C. Some CO gas forms besides hydrocarbon species. There is a very limited number of Si-C duplets.

**Figure 3:**
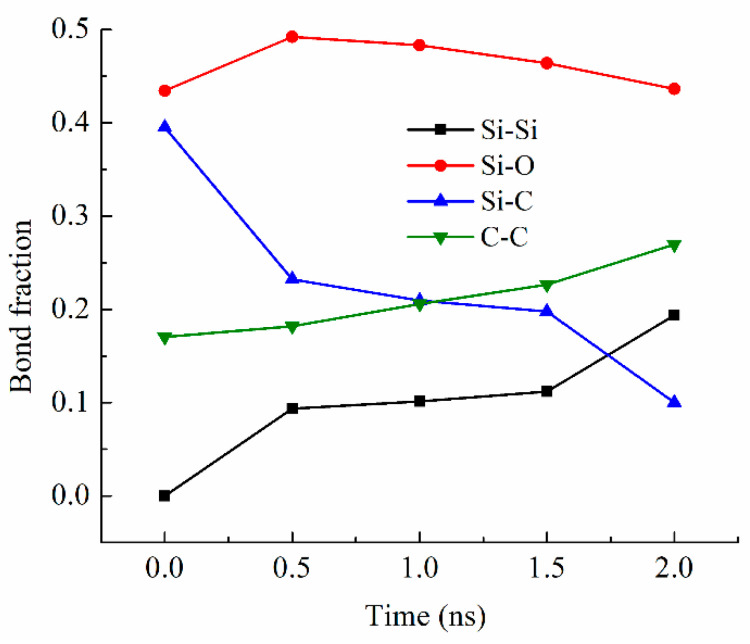
Bond fractions of the PVMS system at different times during 2100 K pyrolysis.

Bond fraction, representing the bond number for a specific type to the total number of bonds in the system, evolves dynamically with temperature. In this work, bonds are defined based on atomic proximity and bonding criteria derived from the ReaxFF potential, which allows for reactive bond-breaking and bond-forming events to be captured accurately [26,27]. The bond types analyzed include Si-O, Si-Si [36,37], Si-C, and C-C, representing key interactions in the system during pyrolysis. Figure 3 shows the changes in the Si-related and C-related bond fractions with the simulation time at 2100 K. The Si-O bond has the highest content, varying from 0.43 to 0.49 (in bond fraction). There is an increase and then a slight decrease due to the changes in other bonds, especially the Si-C bond decrease, which is most drastic and decreases from 0.40 to 0.10 from 0 ns to 2.0 ns of simulation time. The simulation indicates that this Si-C bond decrease is a direct result of the methyl and vinyl group separation from the Si-O backbone. At the same time, the C-C bond fraction steadily increases from 0.17 to 0.27. Collectively, this means that the C-side group decomposition from the Si-O backbone leads to C-rich regions. Also, the Si-Si bond content steadily increases from 0 to 0.19 as the simulation time increases from 0 to 2.0 ns. The Si-Si bond formation is a new observation in the ReaxFF simulation, in this work and our earlier studies [26,27,28,38]. One explanation is that the Si-O clusters are highly active after the C side group separation. The Si-O backbone species have a high tendency to bond among themselves and reduce the system energy. When O atoms are absent from the surrounding regions or Si-O bonds are configurationally unfavorable, Si-Si bond forms. The Si-O bond energy is ~780–785 kJ/mol. The Si-Si bond energy is approximately 208–213 kJ/mol. The Si-Si bond is measured at 2.46 Å and in the second-nearest-neighbor, indicating the large proximity of Si atoms to each other. Thus, Si-Si is a weaker bond, but they can still form when O is absent.

### 3.2. Pyrolysis Temperature Effect

**Figure 4:**
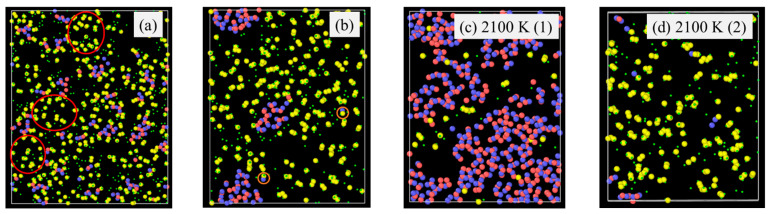
Atomic structure evolution with pyrolysis temperature after 2 ns simulation for PDES system: (**a**) 1500 K, (**b**) 1800 K, (**c**) 2100 K, location (1), and (**d**) 2100 K, location (2). Color scheme: Si, 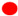; O, 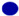; C, 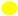; H, 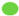.

Pyrolysis temperature has a similar effect on the SiOC atomic structure evolution as the simulation time by affecting bond energies. For example, Si-H (~300–305 kJ/mol) and Si-C (~417–422 kJ/mol) have very different bond energy compared to that of Si-O bond (~780–785 kJ/mol) bonds [39,40,41]. The weaker Si–H and Si-C bonds break more readily, leading to the release of hydrocarbons and the separation of C from SiO_x_ phases. In contrast, the Si–O bond, being significantly stronger, remains stable and contributes to the formation of Si-O clusters. A higher pyrolysis temperature leads to more Si-O and C domain separation, even though the extent varies, depending on the specific precursor system. To illustrate the specific changes, PDES is used as an example as shown in Figure 4. After 2 ns simulation time at 1500 K (Figure 4a), some Si-O clusters form and grow from being strand-like to being equiaxed. C-rich domains can be observed (red circles in Figure 4a). However, there is no large domain separation. Some Si-O clusters are still bonded to C species. At 1800 K simulation, Si-O clusters and C domains separate cleanly. There is no Si in the C domain, which has grown into a large region. Only very few O atoms are still bonded to C (as highlighted by the two small orange circles in Figure 4b). There are no C atoms in the Si-O clusters. With the simulation temperature increase to 2100 K (Figure 4c,d), almost complete Si-O separation from C occurs. Depending on the slicing plane in the 3D simulation volume, almost pure Si-O domains (Figure 4c) or pure C domains (Figure 4d) may be obtained. Figure 4c shows mostly Si-O clusters with a small number of C atoms scattered in between. Slicing farther into the Si-O cluster domain can result in pure Si-O compositions. In Figure 4d, it is mostly C species with a few Si-O clusters. The simulation also shows that a small amount of O atoms continues to bond with the C species. Again, slicing farther into the C cluster domain can result in pure C composition.

**Figure 5:**
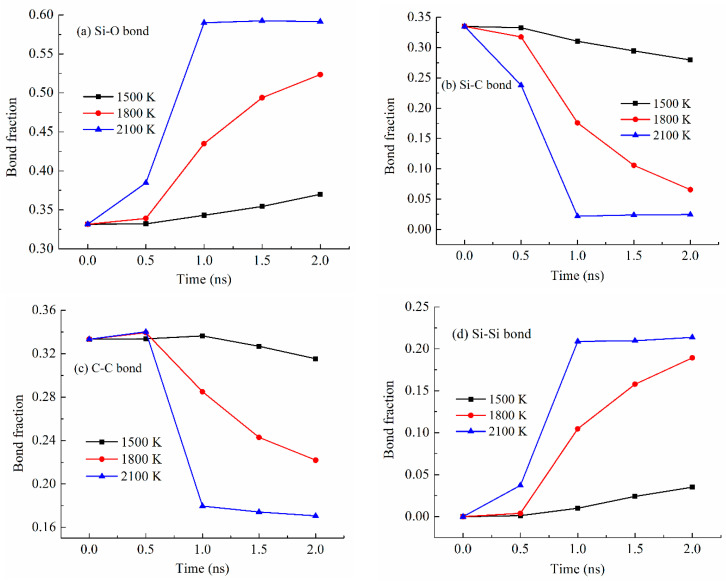
Bond fraction of the pyrolyzing PDES system at different holding times at 1500 K, 1800 K, and 2100 K. (**a**) Si-O bond, (**b**) Si-C bond, (**c**) C-C bond, and (**d**) Si-Si bond.

Different bond fractions evolve simultaneously with the simulation temperature for the PDES system (Figure 5), which is also the case for the other systems. As shown in Figure 5a, with the pyrolysis time increase, the Si-O bond fraction consistently increases. The pyrolysis temperature significantly affects the degree of increase. At 1500 K pyrolysis temperature, the Si-O bond fraction gradually increases, from 0.33 to 0.37, which means that other bonds in the pyrolyzing system are largely present. At 1800 K pyrolysis temperature, the Si-O bond fraction remains steady at 0.33 from 0 to 0.5 ns simulation time and then quickly increases from 0.34 to 0.52. Obviously, a higher pyrolysis temperature of 1800 K is causing drastic changes in Si-C bond, C-C bond, and Si-Si bond. At 2100 K pyrolysis temperature, the Si-O bond fraction quickly increases from 0.33 to 0.59 and then stabilizes at 0.59. This means that 2100 K pyrolysis temperature can cause significant Si-C bond breaking and Si-O bond re-formation during pyrolysis. After the bonds re-form, they remain stable.

Figure 5b shows the Si-C bond fraction changes with the simulation time at different pyrolysis temperatures. In all cases, the Si-C bond fraction decreases with the pyrolysis time, demonstrating the C side group separation from the Si-O backbone. At 1500 K, the Si-C bond fraction decreases slightly from 0.34 to 0.28, due to the lower pyrolysis temperature. At 1800 K, the Si-C bond fraction decreases significantly from 0.34 to 0.065 after a slight decrease in the early period of the holding time. At 2100 K, the Si-C bond fraction decreases significantly starting from 0.34 to 0.022 and then stabilizes at 0.024. The Si-C bond fraction changes are the opposite of what has been observed for the Si-O bond (Figure 5a). This is as expected since the Si-C bond fraction decrease is being compensated for by the Si-O bond fraction increase.

Figure 5c shows the C-C bond fraction change with the simulation time at different pyrolysis temperatures. Surprisingly, there is a C-C bond fraction decrease at all the simulation temperatures. Longer simulation time leads to more C-C bond fraction decrease. In combination with Figure 5b, it means that the separated C side group does not form C-C bonds or domains. Instead, it indicates that the hydrocarbon species have escaped from the pyrolyzing system. This change is especially obvious at higher pyrolysis temperatures. The specific changes can be correlated with the gas release as discussed in Section 3.4.

Figure 5d shows the Si-Si bond fraction increases with the simulation time and temperature. At 1500 K, there is a slight Si-Si bond fraction increase from 0 to 0.035. With the simulation temperature increase to 1800 K, the Si-Si bond fraction significantly increases from 0 to 0.19. At 2100 K simulation temperature, the Si-Si bond fraction increases from 0 to 0.21 and then stabilizes at 0.21. The results in Figure 5d are consistent with the Si-O bond changes in Figure 5a. Higher pyrolysis temperature and longer pyrolysis time lead to more Si-Si bonding to stabilize the pyrolyzing system. Again, this is the first simulation study that has quantified the bond content changing trend with the pyrolysis temperature. It is consistent with our earlier radial distribution function study that reported the Si-Si bond presence along with the C-C bonds [27].

### 3.3. Polymer Structure Effect on SiOC Cluster and C Domain Formation

Bond fraction is a useful parameter to understand the overall bond changes. However, it cannot reveal specific atomic structure evolution. Thus, the understanding of the local atomic structure provided by the ReaxFF simulation can provide important insight in this regard. As shown in Figure 1, among the five precursor systems studied, PDMS has the smallest side group, except for the -H side group in PHMS, which was used to adjust the C content of the PVMS and PMPS systems. All the bonds in the side group of PDMS are saturated. PVMS has a methyl group and a vinyl group (with a C double bond), which affects not only the C amount but also the bond strength between Si and C as well as between C atoms themselves. PDES has a fully saturated ethyl side group, which is different from the vinyl side group in PVMS considering the nature of the bonds between C atoms. PMPS has a methyl and a phenyl group, with the latter being bulky and difficult to escape from the pyrolyzing system. Because of these side group differences, their separation from the Si-O backbone is also different during pyrolysis. This leads to drastically different ceramic atomic evolution.

**Figure 6:**
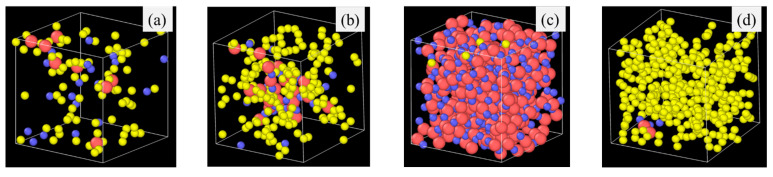
Atomic structures of different polymer precursors after 2100 K pyrolysis for 2 ns: (**a**) PDMS, (**b**) PVMS, (**c**), PDES, and (**d**) PMPS. Color scheme: Si, 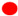; O, 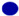; C, 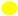.

Since lower pyrolysis temperature leads to less atomic evolution, the atomic structures for the four precursor systems after 2100 K pyrolysis are given in Figure 6. The 3D atomic structures for PDMS, PVMS, PDES, and PMPS systems at various simulation temperatures are given in the supplement (Figures S10 and S11 for PVMS, Figures S12–S14 for PDMS, Figures S15–S17 for PDES, and Figures S18 and S19 for PMPS). Figure 6a shows that the PDMS system has a largely homogeneous SiOC structure. There is limited composition separation. Even in the Si-O rich clusters, C atoms are abundant. Many O atoms bond with C atoms. This means that the methyl side group in PDMS does not separate from the Si-O backbone easily. For PVMS (Figure 6b), however, significant composition separation occurs. There are almost no C atoms that bond with Si. A smaller number of O atoms bond with C. The vinyl group experiences significant separation from the Si-O backbone during pyrolysis. This even causes the methyl group to separate from the Si-O backbone. For PDES (Figure 6c), a large Si-O domain forms and separates from the C-rich regions. There are very few C atoms in such Si-O clusters. The more significant composition and cluster separation is consistent with the vinyl side group effect on PVMS. For PMPS (Figure 6d), C forms many strands (graphitic C in 3D). The Si-O species are sparely dispersed in the C-rich regions. Limited O atoms are bonded to C atoms. This means that the phenyl group separates easily from the Si-O backbone and forms C clusters from its 6-C rings.

In general, Figure 6 shows that fully saturated bonds from the C side groups and larger side groups lead to easier composition separation, as is the case for PDES and PMPS in this study. When the fully bonded C side group is small, Si-O strands cluster together and form a Si-O dominant structure. When the side group is large with a high C content (such as the phenyl group), C atoms cluster into large C domains and further evolve into graphitic C. A side group with saturated C bonds leads to easier composition separation than that with unsaturated C bonds (Figure 6c for PDES vs. Figure 6b for PVMS). The methyl group in Figure 6a is the smallest, which leads to the most homogeneous composition distribution in SiOC. It also offers a higher probability for O atoms to bond with C atoms.

### 3.4. Gas Release Difference

**Figure 7:**
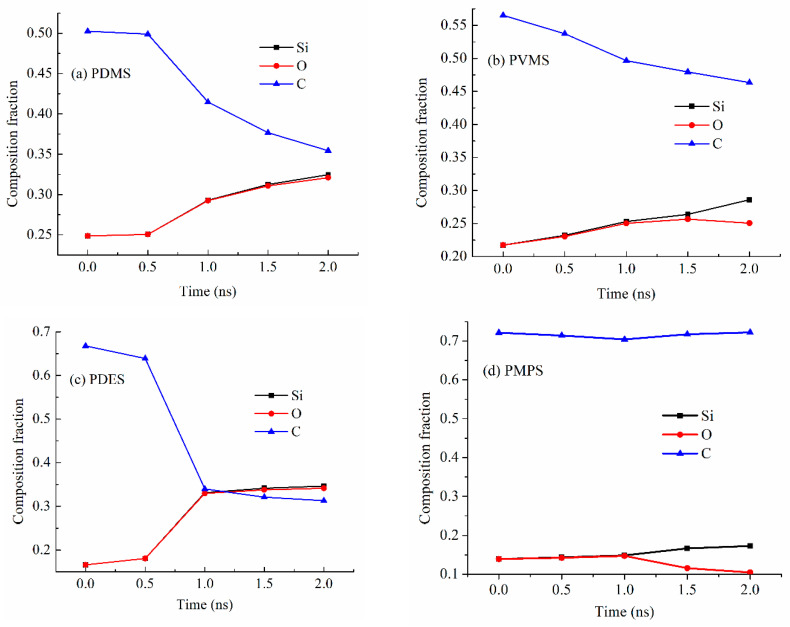
Compositions (atomic fractions) of the pyrolyzing (**a**) PDMS, (**b**) PVMS, (**c**) PDES, and (**d**) PMPS systems at different simulation time points at 2100 K.

Figure 7a shows the Si, O, and C composition changes for the PDMS precursor system at 2100 K. The Si and O contents are almost the same, with the atomic fraction increasing from 0.25 to 0.32. The overlapping curves for Si and O in Figure 7a also mean that there is little O loss during the pyrolysis process since Si is not evaporative. However, the C content decreases significantly from 0.50 to 0.35 with the pyrolysis time increase from 0.5 ns to 2.0 ns, meaning that all the C loss has been through hydrocarbon or CO/CO_2_ loss. This means that the methyl side group separation from the Si-O backbone is the dominant mechanism for the PDMS decomposition and SiOC formation.

Figure 7b shows the Si, O, and C content changes for the PVMS precursor system at 2100 K. The Si and O contents are almost the same, increasing from 0.22 to 0.26, until 1.5 ns holding time, then there is a Si content increase to 0.29 and an O content decrease to 0.25, indicating that the PVMS precursor starts to lose O during the pyrolysis process. There is also a steady C content decrease from 0.57 to 0.46 during the 2.0 ns pyrolysis time. The simultaneous C and O content decreases indicate CO/CO_2_ gas formation and evaporation. Since the vinyl group is believed to separate from the Si-O backbone first [27], which means that the methyl groups are being oxidized after their separation from the Si-O backbone starting at 1.5 ns. However, the overall C content decrease is not as high as for the PDMS system. This means that more C atoms are forming C-C domains.

Figure 7c shows the Si, O, and C content changes for the PDES precursor system at 2100 K. The simulation results show that the Si and O contents have the same changes, increasing from 0.17 to 0.34, indicating that there is little O loss during the pyrolysis process. The content increases are due to the C content decrease. Also, the Si and O content increase from 0.17 to 0.34 and the C content decrease from 0.67 to 0.31 occur simultaneously, in opposite directions. This means that most C atoms are lost through hydrocarbon loss and CO/CO_2_ loss is negligible. The significant and early loss of C atoms also means that the ethyl group separates from the Si-O backbone easily.

Figure 7d shows the Si, O, and C content changes for the PMPS precursor system at 2100 K. The Si and O contents are almost the same until 1.0 ns pyrolysis time, increasing from 0.14 to 0.15, then there is a Si content increase and an O content decrease, indicating that it starts to lose O through CO/CO_2_ during the pyrolysis process. Since the O loss occurs in the later stage of the holding time, the simulation results indicate that the C atoms from the methyl group are oxidized by O species. Considering that most of the C content in PMPS comes from the phenyl group, the overall C content is steady, at ~0.71. This means that most C atoms contribute to the formation of C-C domains.

**Figure 8:**
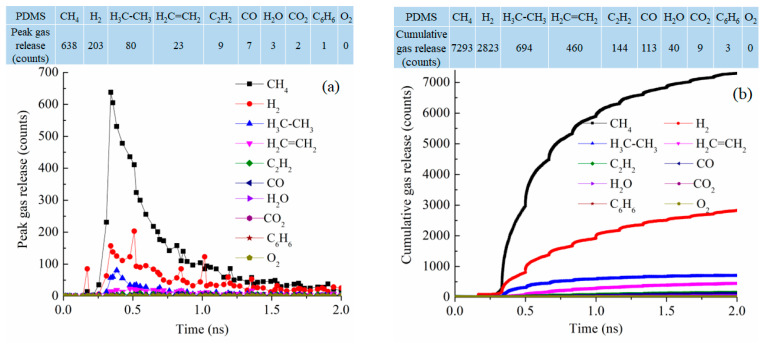
Gas release results for the PDMS pyrolyzing system at 2100 K: (**a**) gas release count as a function of simulation time, (**b**) cumulative gas release count as a function of simulation time.

Since the simulation systems have similar sizes (from 84,352 atoms to 102,912 atoms), the pyrolysis process can also be examined from the type and number of released gas molecules as a function of pyrolysis time. Figure 8 shows the gas release results for the PDMS system at 2100 K. In the early stage of the pyrolysis, around 0.4 ns, CH_4_ release is the most significant, followed by H_2_ and then C_2_H_6_ and C_2_H_4_ [42,43]. The peak release is 638 molecules for CH_4_, 203 molecules for H_2_, 80 molecules for C_2_H_6_, and 23 molecules for C_2_H_4_ while the release of the other gas species is in the single digit or 0, as given in the table in Figure 8a. This means that the methyl groups are released along with polymer condensation and re-bonding and formation of H_2_, C_2_H_6_, and C_2_H_4_. This is correspondingly represented by the cumulative gas release results in Figure 8b. The total release is 7293 molecules for CH_4_, 2823 molecules for H_2_, 694 molecules for C_2_H_6_, and 460 molecules for C_2_H_4_. The dominant gas species are CH_4_ and H_2_. There are little or no O-containing species such as CO, CO_2_, O_2_, or H_2_O. C_6_H_6_ release is also almost none. Since the PDMS precursor has two methyl side groups in each repeating unit, the results indicate that the precursor decomposition is mainly through methyl group detachment and evolution. The total gas molecules released are 11,579.

**Figure 9:**
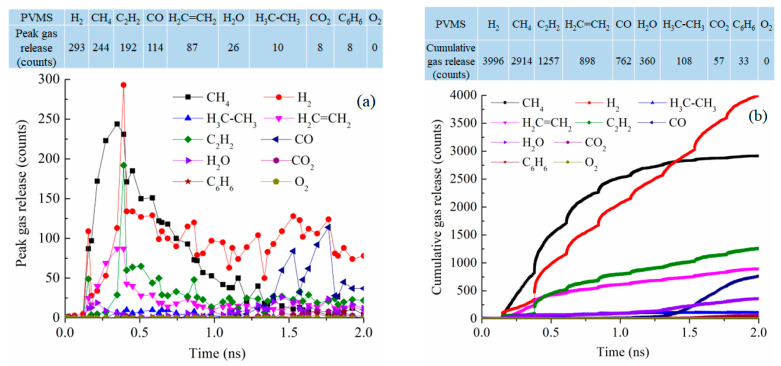
Gas release results for the PVMS pyrolyzing system at 2100 K: (**a**) peak gas release counts, (**b**) cumulative gas release counts.

For the PVMS system at 2100 K pyrolysis, the gas release process is spread out throughout the simulation instead of being concentrated in a certain stage (Figure 9a). CH_4_ and H_2_ releases are still the most significant with 244 and 293 molecules, respectively, as the peak values around 0.35–0.39 ns. C_2_H_2_ loss is also significant at 192 molecules. This means that the methyl group detachment from the Si-O backbone is most likely, followed by polymer condensation and vinyl group detachment. However, the polymer decomposition is a sluggish process throughout the simulation. This is reflected not only in the prolonged process of gas release but also in the small numbers of removed gas molecules. Compared to the PDMS system, the difference is clear in the number of released CH_4_ with only 38% of that from the PDMS pyrolysis at peak release time. Figure 9b shows that CH_4_ release is faster than other gasses in the early stage of the pyrolysis process. Then, H_2_ gas release keeps increasing until it surpasses CH_4_ release at ~1.5 ns. The overall CH_4_ and H_2_ release is much lower than that from the PDMS system. There is also increasing C_2_H_2_ and C_2_H_4_ removal. This means that the methyl group detachment happens first, followed by the vinyl group removal. After 1.35 ns simulation, there is an increase in CO gas release. This O loss is due to the disintegration of the Si-O backbone. Not surprisingly, there is a small amount of CO_2_ and even some C_6_H_6_ molecules. Understandably, there is no loss of O_2_. The total released gas molecules are 10,385, about 1200 less than that of the PDMS system.

**Figure 10:**
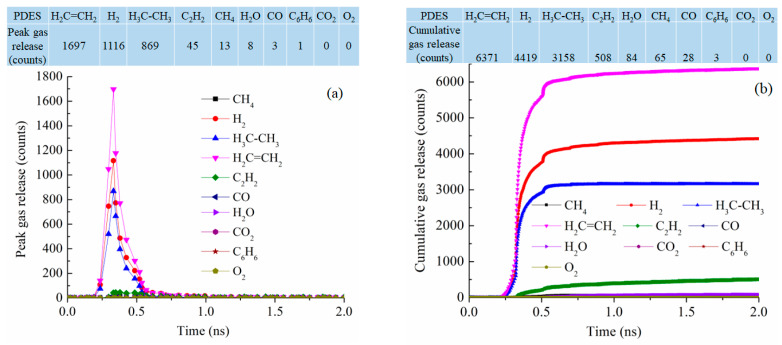
Gas release results for the PDES pyrolyzing system at 2100 K: (**a**) peak gas release counts, (**b**) cumulative gas release counts.

At 2100 K pyrolysis temperature, PDES has the best-defined gas release behavior, with narrowly defined peaks for C_2_H_4_, H_2_, C_2_H_6_, and C_2_H_2_ at 0.33 ns simulation time, the numbers of peak gas molecules released are 1697, 1116, 869, and 42 (Figure 10a). This means that the ethyl groups are mostly removed as C_2_H_4_ and C_2_H_6_ and the polymer precursor molecules also condense to release a high number of H_2_ molecules (1116). This also explains why PDES has the largest C loss in Figure 7c. The cumulative gas release is also well defined with the gas release reaching the plateau values for C_2_H_4_, H_2_, C_2_H_6_, and C_2_H_2_ at 6371, 4419, 3158, and 508 at ~0.75 ns (Figure 10b). There is negligible release of H_2_O, CH_4_, CO, C_6_H_6_, CO_2_, and O_2_. This means that the ethyl group has a clean and consistent separation behavior from the Si-O backbone. Understandably, there is no CH_4_ or C_6_H_6_ loss as the molecular structure has no methyl or phenyl group. Also, there is almost no O removal from the Si-O backbone. The total gas molecules lost are 14,636, the highest out of the four polymer precursor systems studied.

**Figure 11:**
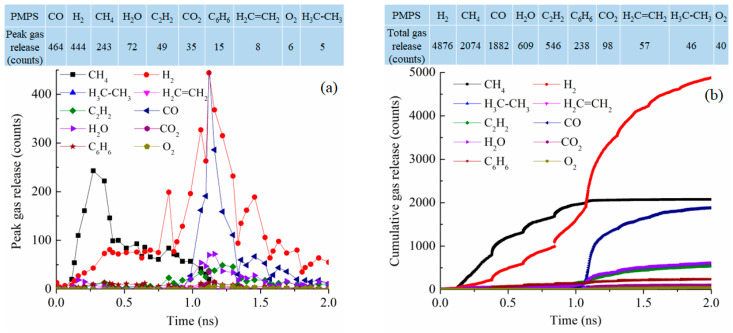
Gas release results for the PMPS pyrolyzing system at 2100 K: (**a**) peak gas release counts, (**b**) cumulative gas release counts.

The gas release for the PMPS system at 2100 K has a bi-modal distribution as shown in Figure 11a. At 0.3 ns, CH_4_ reaches its peak gas release number of 243. At 1.12 ns, CO and H_2_ reach the peak numbers of 464 and 444, respectively. These gas release curves are not well defined. Cumulatively, as shown in Figure 11b, the total gas release is highest for H_2_, at 4876 followed by CH_4_ at 2074 molecules, CO at 1882 molecules, H_2_O at 609 molecules, C_2_H_2_ at 546 molecules, and C_6_H_6_ at 238 molecules. The methyl group is likely removed as CH_4_, with the polymer condensation leading to H_2_ release. However, the phenyl group is well conserved and is believed to have converted into graphitic C. The total number of gasses released is 10,466, comparable to those of the PDMS and PVMS systems but much lower than that for the PDES system.

Overall, based on our simulation results, PDMS pyrolysis releases CH_4_ the most due to the presence of the only methyl functional group. PVMS releases CH_4_ the most followed by C_2_H_2_, thanks to the presence of both the methyl and vinyl groups. PDES releases C_2_H_4_ and C_2_H_6_, almost exclusively due to its ethyl group presence. PMPS releases CH_4_ because of the methyl group presence; the phenyl group is stable and converted to turbostratic C, remaining in the pyrolyzed ceramic. PMPS pyrolysis also leads to a large amount of CO and noticeable O_2_ releases. For the cumulative gas release, O_2_ counts at 0.06% of the total gas released. This means that the O_2_ species is negligible. It is either in transient states involved in bond re-formation or reduction process. We do not believe that O_2_ leaves the SiOC system as a gaseous product. The above behaviors are consistent with the polymer precursor molecular structure and functional group scission from the Si-O backbone. H_2_ is always released at a high amount for all the precursor systems due to polymer side group breakup and C-C bond redistribution. This simulation effort provides not only the exact sequence of the gas release but also the quantitative data of the released species for each given polymer precursor.

## 4. Conclusions

This study advances a ReaxFF-based molecular dynamic simulation method to understand the polymer-to-ceramic conversion process with different pyrolysis times, temperatures, and precursor molecular structures. Longer pyrolysis time and higher pyrolysis temperature lead to more Si-O and C cluster separation. Larger side groups and fully saturated C side groups lead to easier phase separation with Si-O or C domains. Gas release during pyrolysis has been examined for the first time and shows that methyl and ethyl side groups are released as CH_4_, C_2_H_6_, and C_2_H_4_ along with H_2_ formation. The specific sequences and amounts are related to the molecular structures of the polymer precursors. This study provides the first in-depth understanding of polymer precursor and pyrolysis condition effects on the atomic structure evolution of SiOC ceramic during the early stage of pyrolysis. It provides the much-needed quantitative knowledge of polymer pyrolysis conversion to ceramics.

The novelty of this study lies in several key aspects: advanced ReaxFF-based molecular dynamics simulation, systematic evaluation of pyrolysis conditions, first-time examination of detailed gas release during pyrolysis, insight into phase separation mechanisms, quantitative understanding of early-stage polymer-to-ceramic conversion. This research unravels the coupling of molecular structure, pyrolysis conditions, and gas release mechanisms to uncover new insights into the early-stage atomic structure evolution of SiOC ceramics, which has not been explored in this depth before. These findings offer crucial insights into PSO pyrolysis mechanisms, particularly in SiOC formation, gas release, and bond transformations, aiding the design of SiOC ceramics for a wide range of applications.

## Data Availability

The original contributions presented in the study are included in the article; further inquiries can be directed to the corresponding author.

## Supplementary Materials

The following supporting information can be downloaded at: https://www.mdpi.com/article/10.3390/ma18071412/s1, Figure S1. Atomic structural evolution in 2D view for the PVMS system at 1800 K simulation temperature: (a) 0 ns, (b) 0.5 ns, (c) 1 ns, (d) 1.5 ns, and (e) 2 ns; Figure S2. Atomic structural evolution in 2D view for the PDMS system at 1500 K simulation temperature: (a) 0 ns, (b) 0.5 ns, (c) 1 ns, (d) 1.5 ns, and (e) 2 ns; Figure S3. Atomic structural evolution in 2D view for the PDMS system at 1800 K simulation temperature: (a) 0 ns, (b) 0.5 ns, (c) 1 ns, (d) 1.5 ns, and (e) 2 ns; Figure S4. Atomic structural evolution in 2D view for the PDMS system at 2100 K simulation temperature: (a) 0 ns, (b) 0.5 ns, (c) 1 ns, (d) 1.5 ns, and (e) 2 ns; Figure S5. Atomic structural evolution in 2D view for the PDES system at 1500 K simulation temperature: (a) 0 ns, (b) 0.5 ns, (c) 1 ns, (d) 1.5 ns, and (e) 2 ns; Figure S6. Atomic structural evolution in 2D view for the PDES system at 1800 K simulation temperature: (a) 0 ns, (b) 0.5 ns, (c) 1 ns, (d) 1.5 ns, and (e) 2 ns; Figure S7. Atomic structural evolution in 2D view for the PDES system at 2100 K simulation temperature: (a) 0 ns, (b) 0.5 ns, (c) 1 ns, (d) 1.5 ns, and (e) 2 ns; Figure S8. Atomic structural evolution in 2D view for the PMPS system at 1800 K simulation temperature: (a) 0 ns, (b) 0.5 ns, (c) 1 ns, (d) 1.5 ns, and (e) 2 ns; Figure S9. Atomic structural evolution in 2D view for the PMPS system at 2100 K simulation temperature: (a) 0 ns, (b) 0.5 ns, (c) 1 ns, (d) 1.5 ns, and (e) 2 ns; Figure S10. Atomic structural evolution in 3D view for the PVMS system at 1800 K simulation temperature: (a) 0 ns, (b) 0.5 ns, (c) 1 ns, (d) 1.5 ns, and (e) 2 ns; Figure S11. Atomic structural evolution in 3D view for the PVMS system at 2100 K simulation temperature: (a) 0 ns, (b) 0.5 ns, (c) 1 ns, (d) 1.5 ns, and (e) 2 ns; Figure S12. Atomic structural evolution in 3D view for the PDMS system at 1500 K simulation temperature: (a) 0 ns, (b) 0.5 ns, (c) 1 ns, (d) 1.5 ns, and (e) 2 ns; Figure S13. Atomic structural evolution in 3D view for the PDMS system at 1800 K simulation temperature: (a) 0 ns, (b) 0.5 ns, (c) 1 ns, (d) 1.5 ns, and (e) 2 ns; Figure S14. Atomic structural evolution in 3D view for the PDMS system at 2100 K simulation temperature: (a) 0 ns, (b) 0.5 ns, (c) 1 ns, (d) 1.5 ns, and (e) 2 ns; Figure S15. Atomic structural evolution in 3D view for the PDES system at 1500 K simulation temperature: (a) 0 ns, (b) 0.5 ns, (c) 1 ns, (d) 1.5 ns, and (e) 2 ns; Figure S16. Atomic structural evolution in 3D view for the PDES system at 1800 K simulation temperature: (a) 0 ns, (b) 0.5 ns, (c) 1 ns, (d) 1.5 ns, and (e) 2 ns; Figure S17. Atomic structural evolution in 3D view for the PDES system at 2100 K simulation temperature: (a) 0 ns, (b) 0.5 ns, (c) 1 ns, (d) 1.5 ns, and (e) 2 ns; Figure S18. Atomic structural evolution in 3D view for the PMPS system at 1800 K simulation temperature: (a) 0 ns, (b) 0.5 ns, (c) 1 ns, (d) 1.5 ns, and (e) 2 ns; Figure S19. Atomic structural evolution in 3D view for the PMPS system at 2100 K simulation temperature: (a) 0 ns, (b) 0.5 ns, (c) 1 ns, (d) 1.5 ns, and (e) 2 ns.
