# Supplementary material for: Understanding Polysiloxane Polymer to Amorphous SiOC Conversion During Pyrolysis Through ReaxFF Simulation
Source: Materials (Basel). 2025 Mar 22;18(7):1412. doi: 10.3390/ma18071412 (PMC11989382; doi:10.3390/ma18071412)
Supplement: Supplementary file 1 [file materials-18-01412-s001.zip › materials-3467344-supplementary.pdf]

Supplement

Understanding polysiloxane polymer to amorphous SiOC conversion during pyrolysis through ReaxFF simulation

Kathy Lu,[1,2,*] Harrison Chaney[1]

1. Department of Materials Science and Engineering, Virginia Polytechnic Institute and State University, Blacksburg, Virginia, 24061, USA
2. Department of Mechanical and Materials Engineering, University of Alabama at Birmingham, Alabama, 35294, USA

*Corresponding author: Email: klu@uab.edu

Conflict of interest: None.

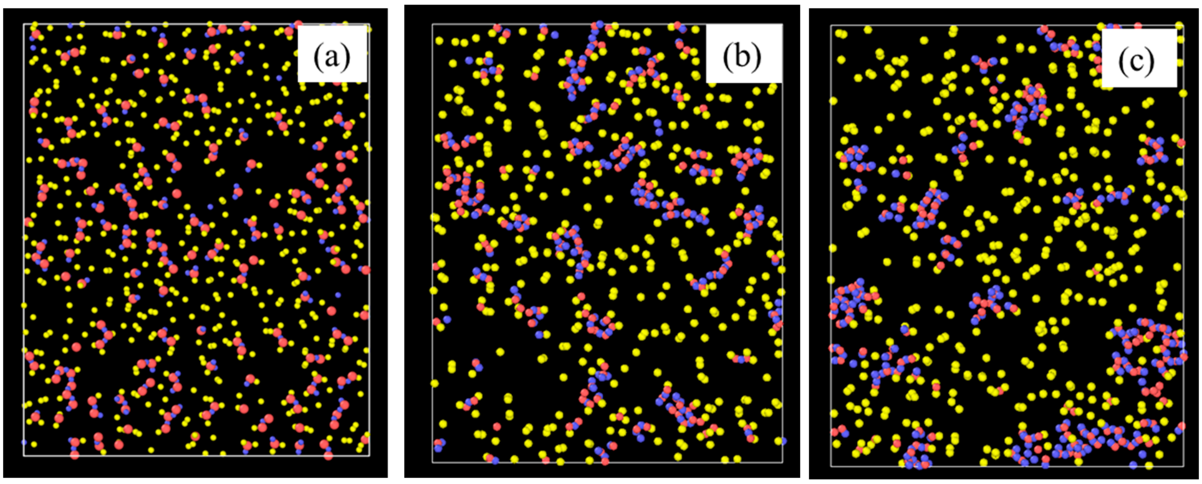

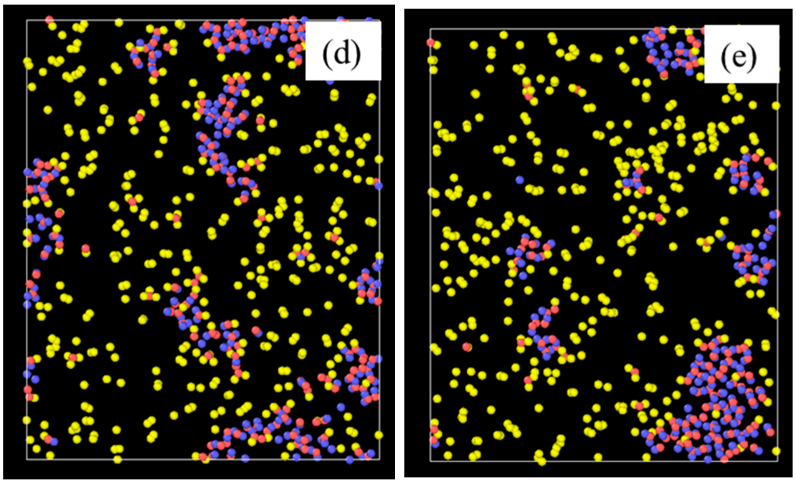

Figure S1. Atomic structural evolution in 2D view for the PVMS system at 1800 K simulation temperature: (a) 0 ns, (b) 0.5 ns, (c) 1 ns, (d) 1.5 ns, and (e) 2 ns.

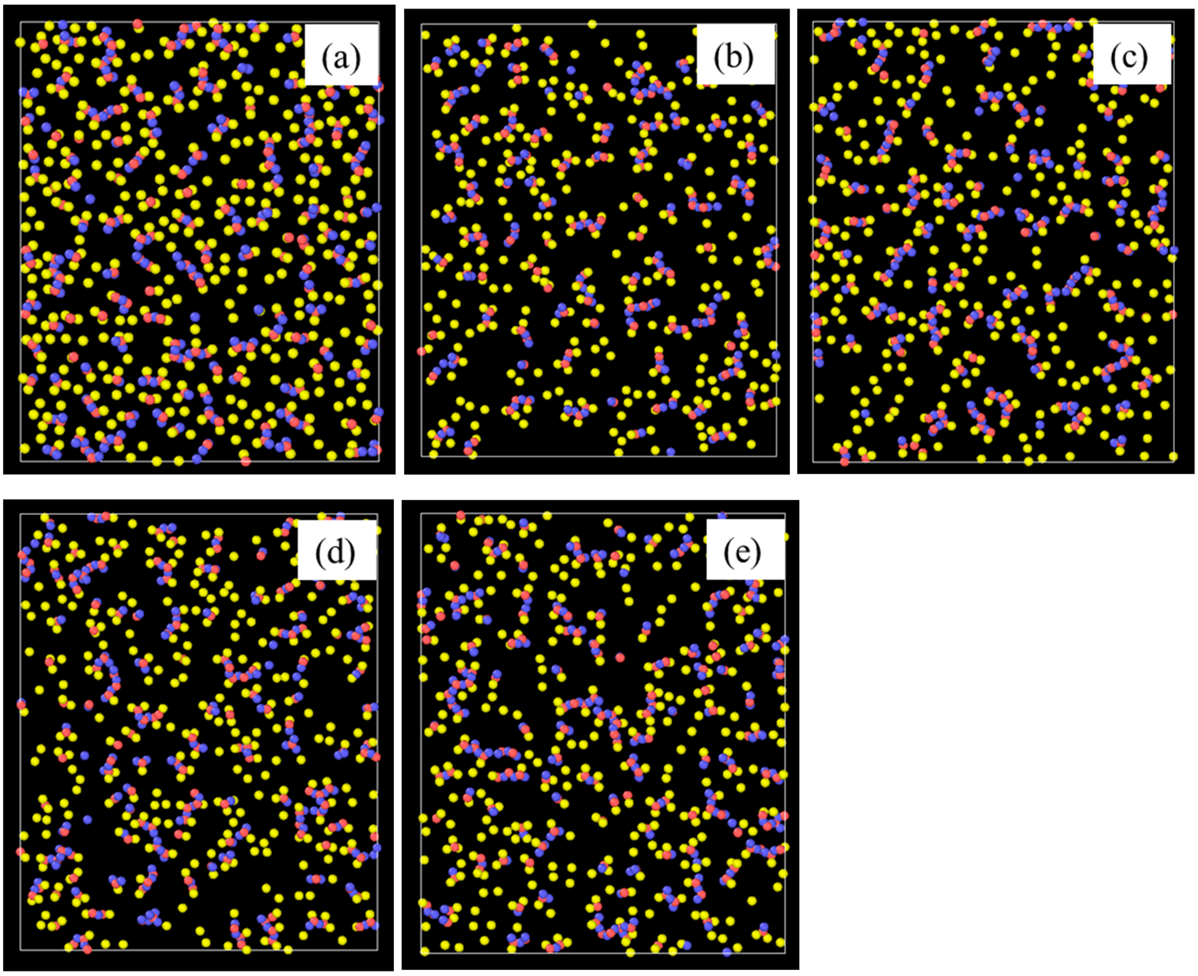

Figure S2. Atomic structural evolution in 2D view for the PDMS system at 1500 K simulation temperature: (a) 0 ns, (b) 0.5 ns, (c) 1 ns, (d) 1.5 ns, and (e) 2 ns.

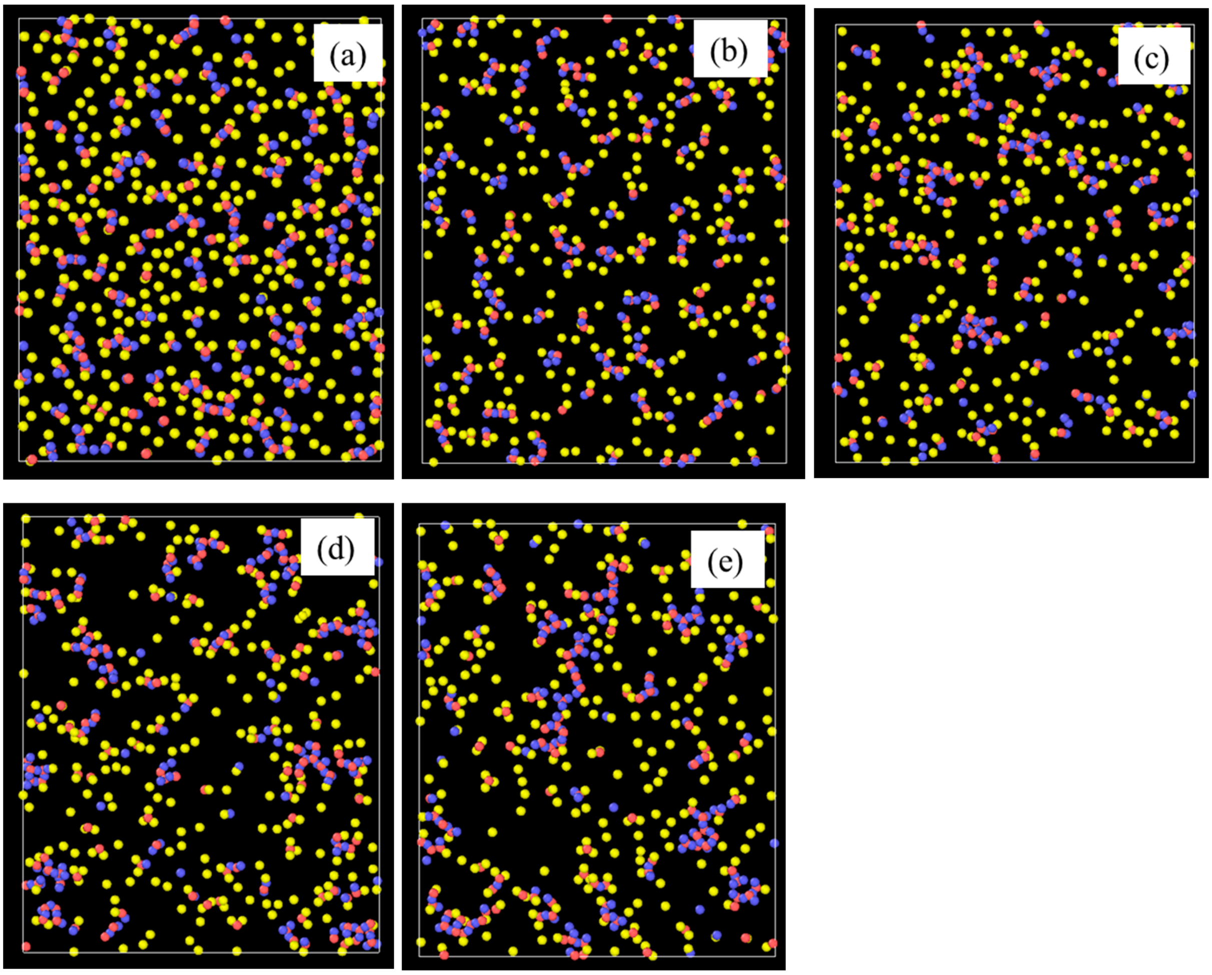

Figure S3. Atomic structural evolution in 2D view for the PDMS system at 1800 K simulation temperature: (a) 0 ns, (b) 0.5 ns, (c) 1 ns, (d) 1.5 ns, and (e) 2 ns.

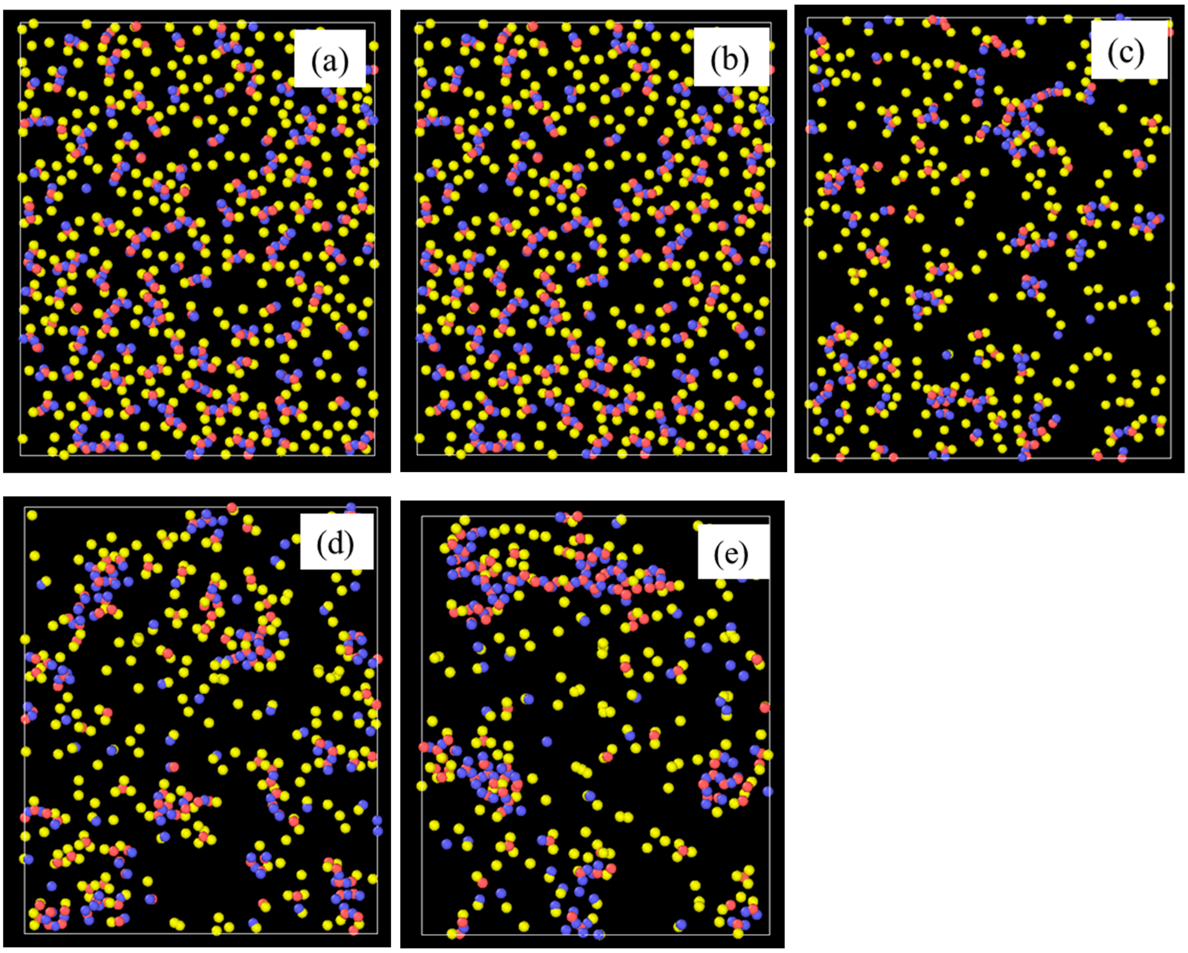

Figure S4. Atomic structural evolution in 2D view for the PDMS system at 2100 K simulation temperature: (a) 0 ns, (b) 0.5 ns, (c) 1 ns, (d) 1.5 ns, and (e) 2 ns.

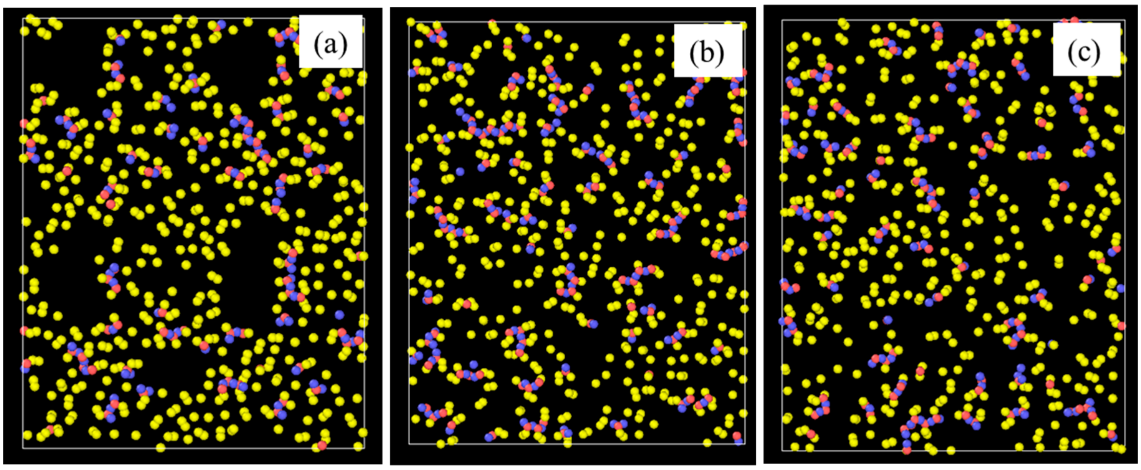

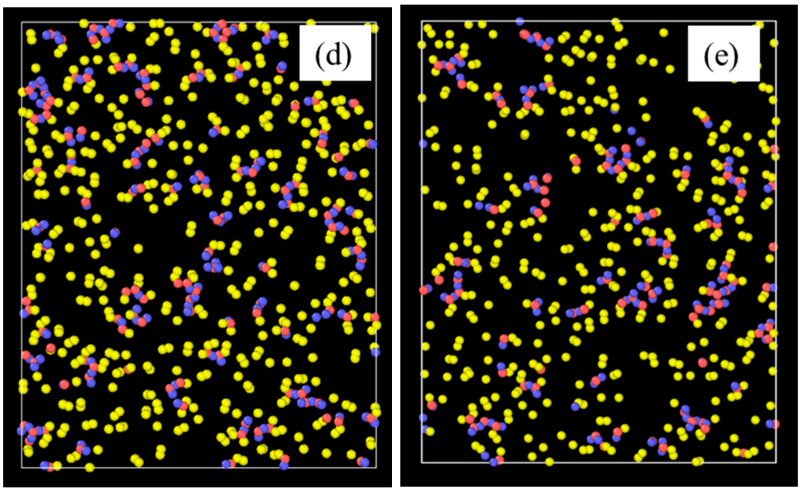

Figure S5. Atomic structural evolution in 2D view for the PDES system at 1500 K simulation temperature: (a) 0 ns, (b) 0.5 ns, (c) 1 ns, (d) 1.5 ns, and (e) 2 ns.

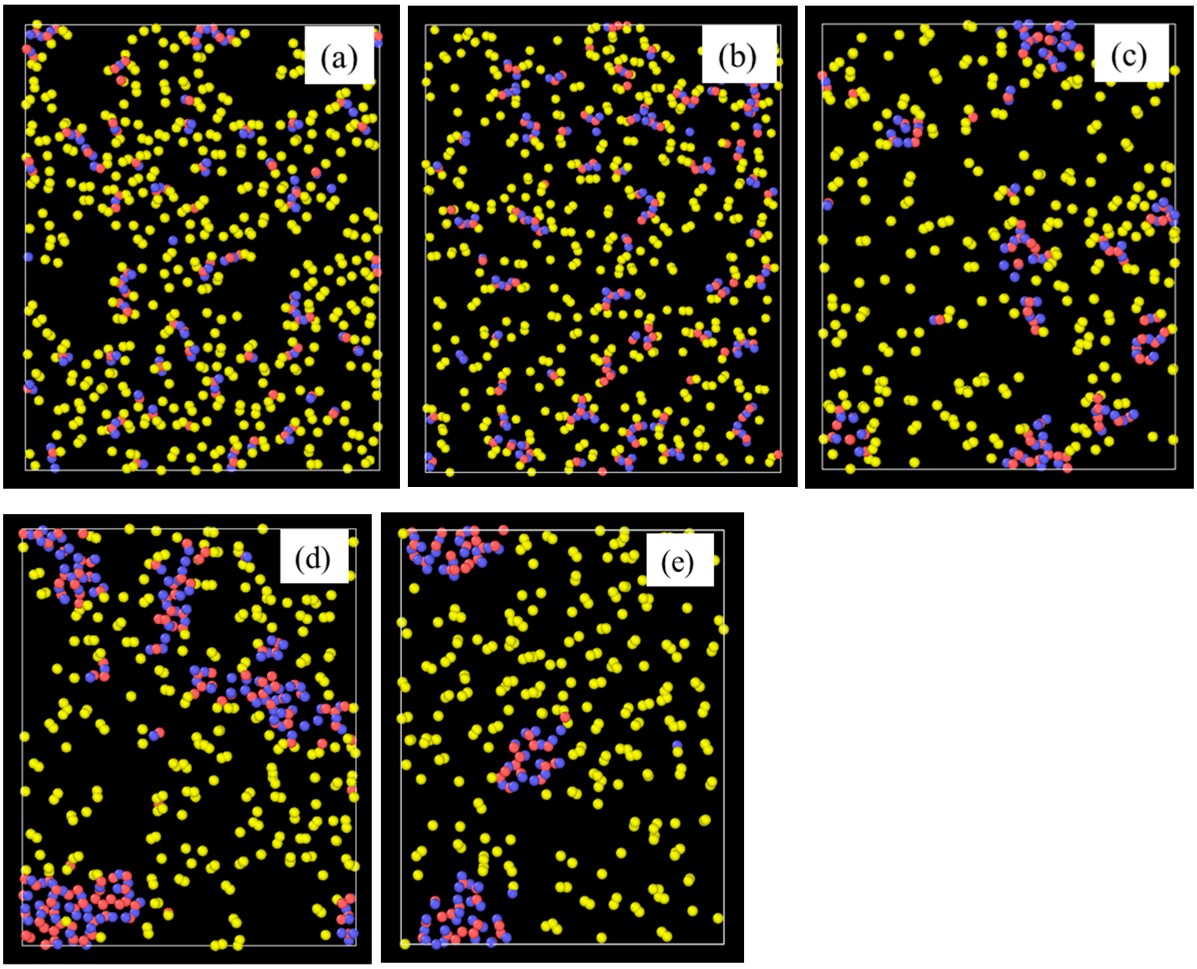

Figure S6. Atomic structural evolution in 2D view for the PDES system at 1800 K simulation temperature: (a) 0 ns, (b) 0.5 ns, (c) 1 ns, (d) 1.5 ns, and (e) 2 ns.

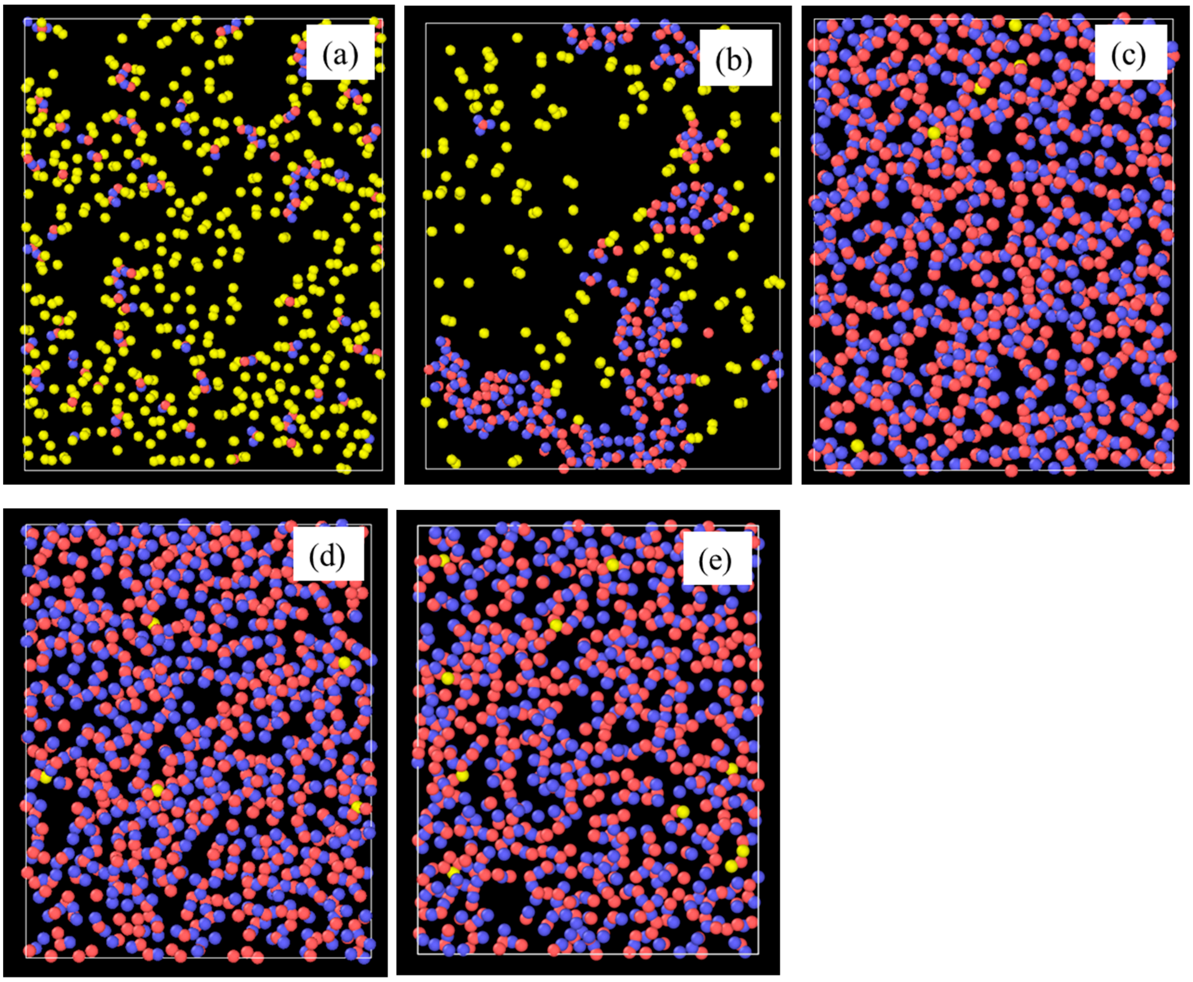

Figure S7. Atomic structural evolution in 2D view for the PDES system at 2100 K simulation temperature: (a) 0 ns, (b) 0.5 ns, (c) 1 ns, (d) 1.5 ns, and (e) 2 ns.

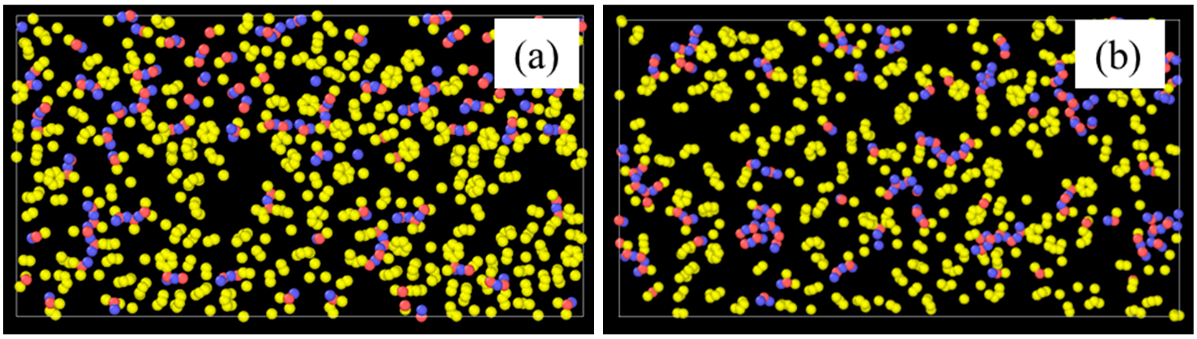

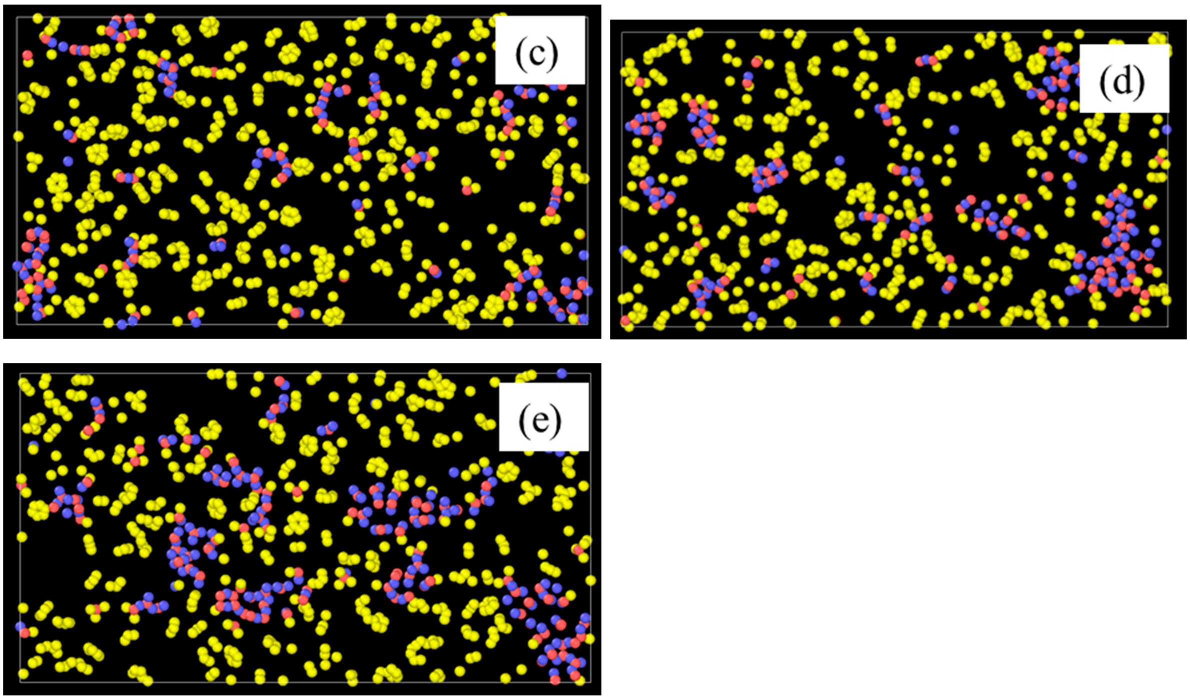

Figure S8. Atomic structural evolution in 2D view for the PMPS system at 1800 K simulation temperature: (a) 0 ns, (b) 0.5 ns, (c) 1 ns, (d) 1.5 ns, and (e) 2 ns.

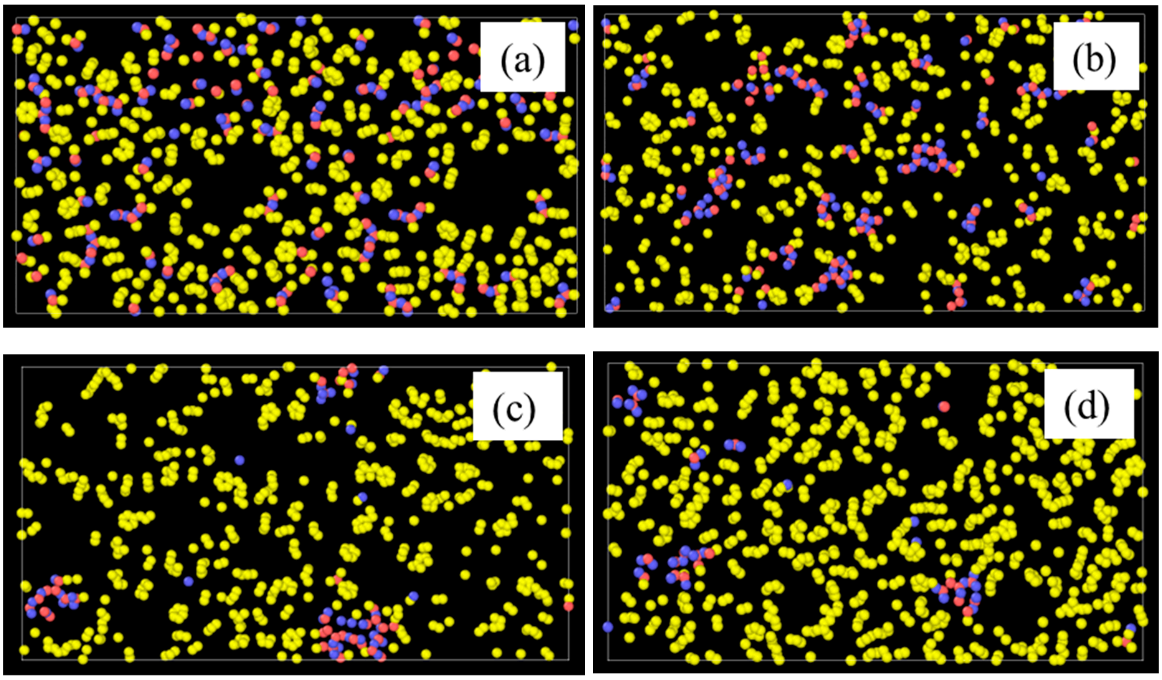

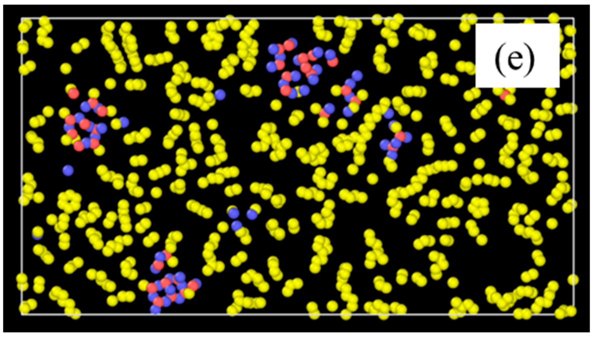

Figure S9. Atomic structural evolution in 2D view for the PMPS system at 2100 K simulation temperature: (a) 0 ns, (b) 0.5 ns, (c) 1 ns, (d) 1.5 ns, and (e) 2 ns.

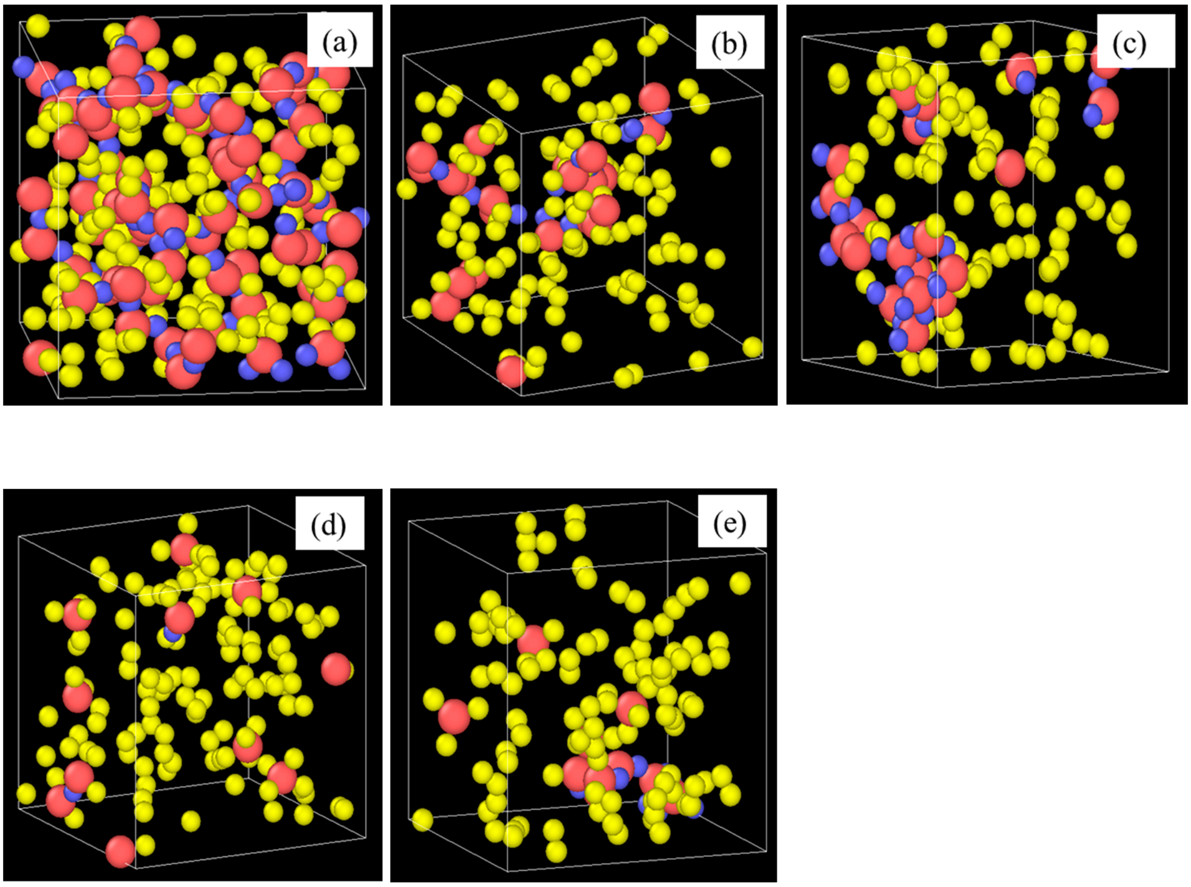

Figure S10. Atomic structural evolution in 3D view for the PVMS system at 1800 K simulation temperature: (a) 0 ns, (b) 0.5 ns, (c) 1 ns, (d) 1.5 ns, and (e) 2 ns.

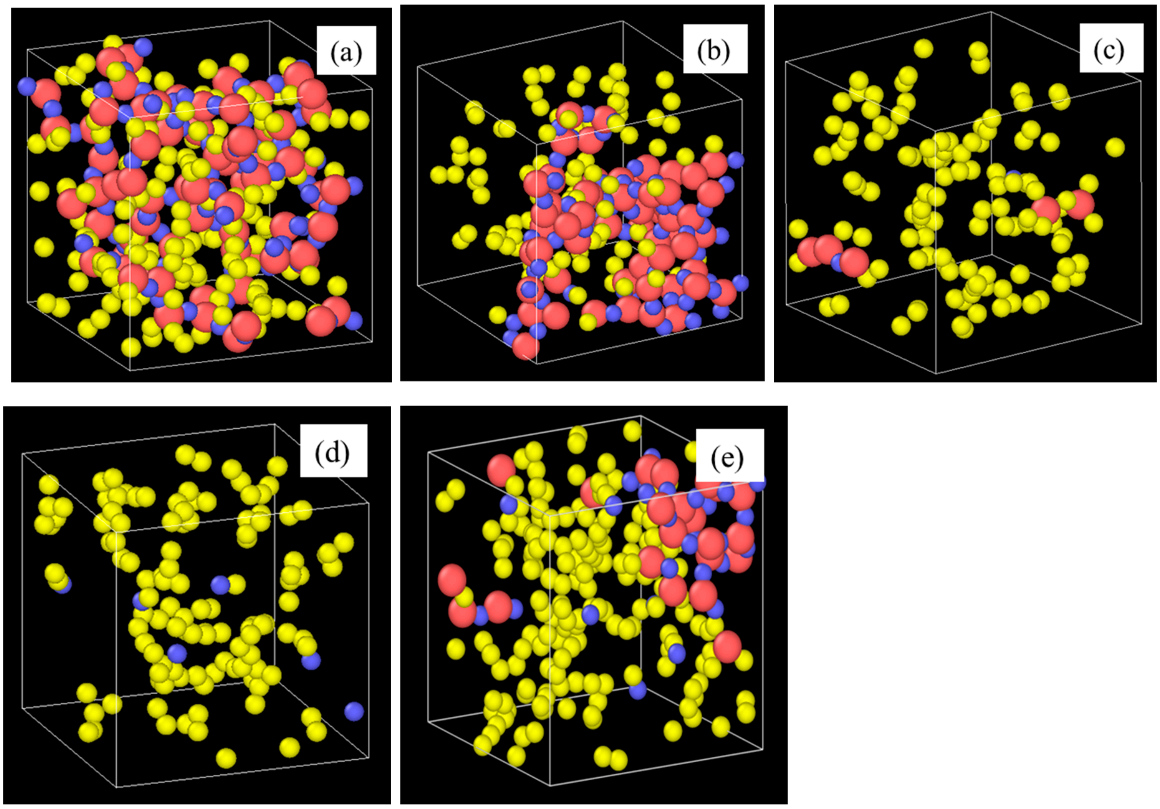

Figure S11. Atomic structural evolution in 3D view for the PVMS system at 2100 K simulation temperature: (a) 0 ns, (b) 0.5 ns, (c) 1 ns, (d) 1.5 ns, and (e) 2 ns.

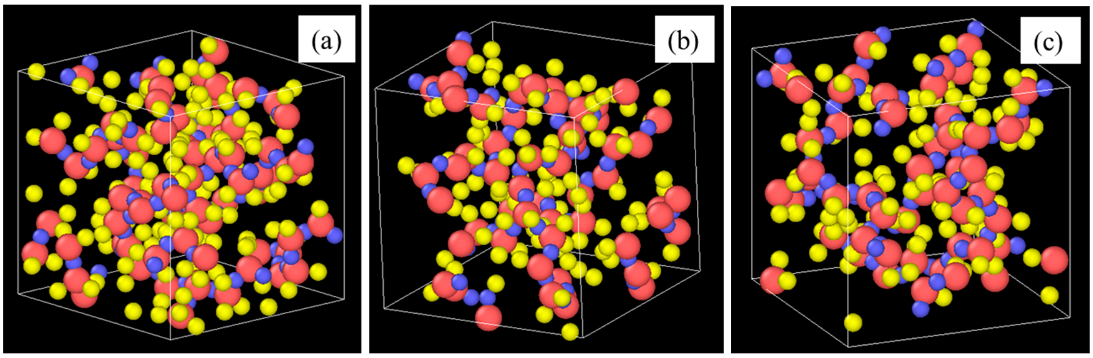

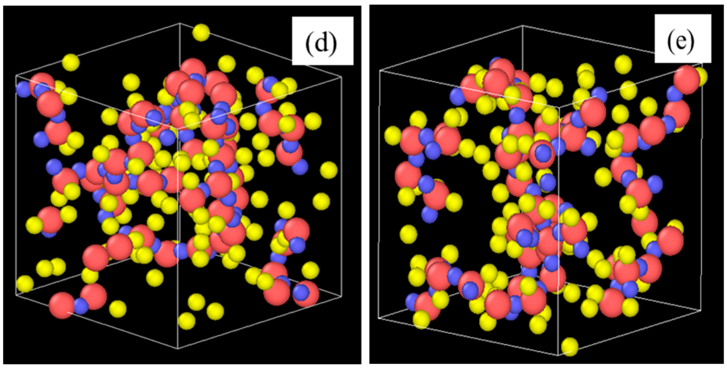

Figure S12. Atomic structural evolution in 3D view for the PDMS system at 1500 K simulation temperature: (a) 0 ns, (b) 0.5 ns, (c) 1 ns, (d) 1.5 ns, and (e) 2 ns.

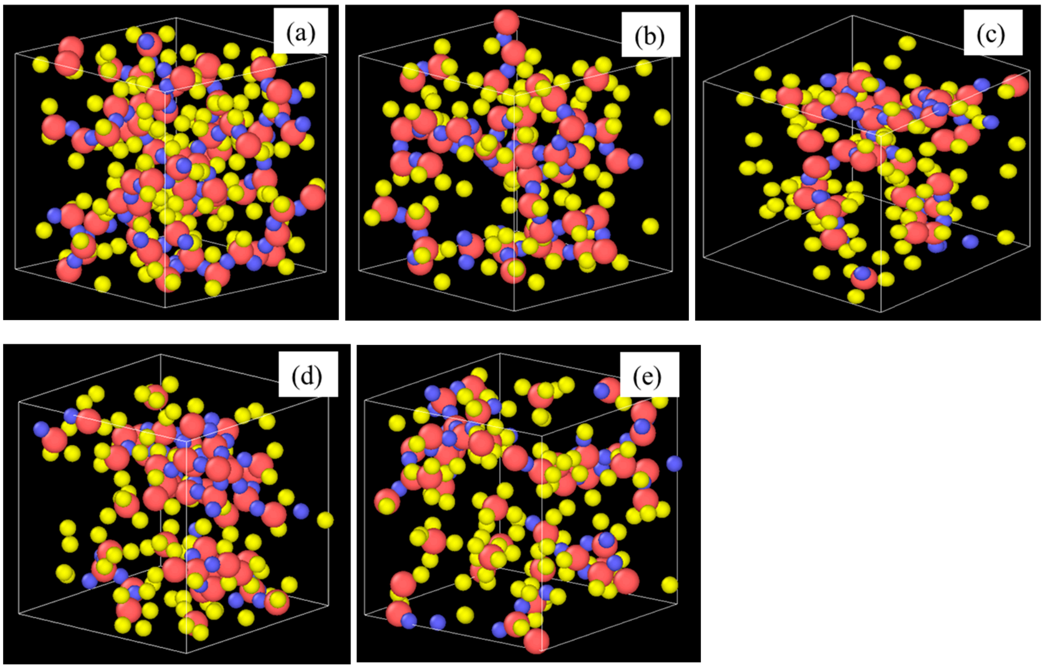

Figure S13. Atomic structural evolution in 3D view for the PDMS system at 1800 K simulation temperature: (a) 0 ns, (b) 0.5 ns, (c) 1 ns, (d) 1.5 ns, and (e) 2 ns.

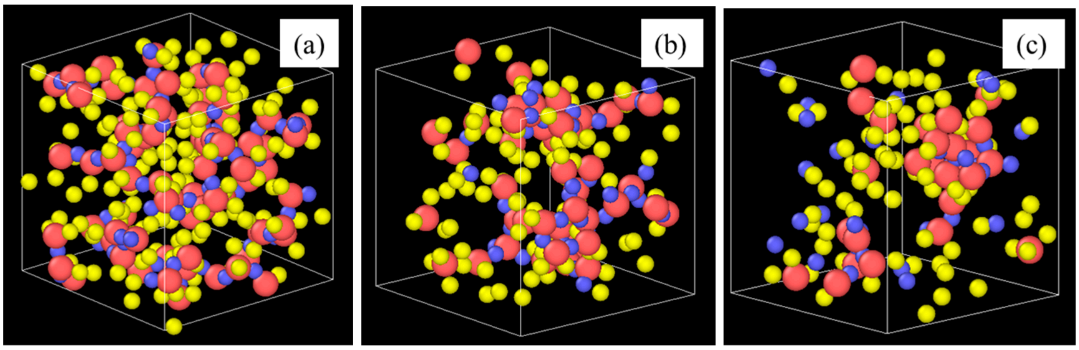

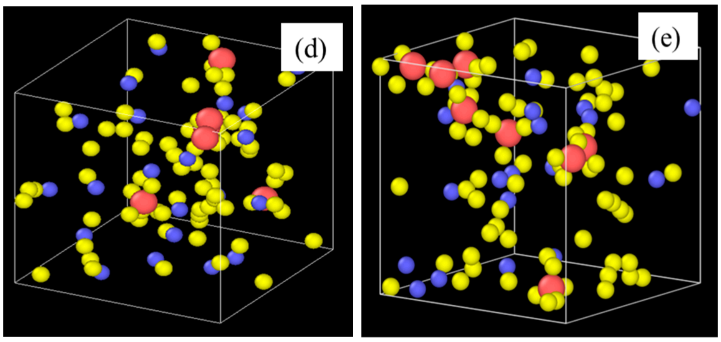

Figure S14. Atomic structural evolution in 3D view for the PDMS system at 2100 K simulation temperature: (a) 0 ns, (b) 0.5 ns, (c) 1 ns, (d) 1.5 ns, and (e) 2 ns.

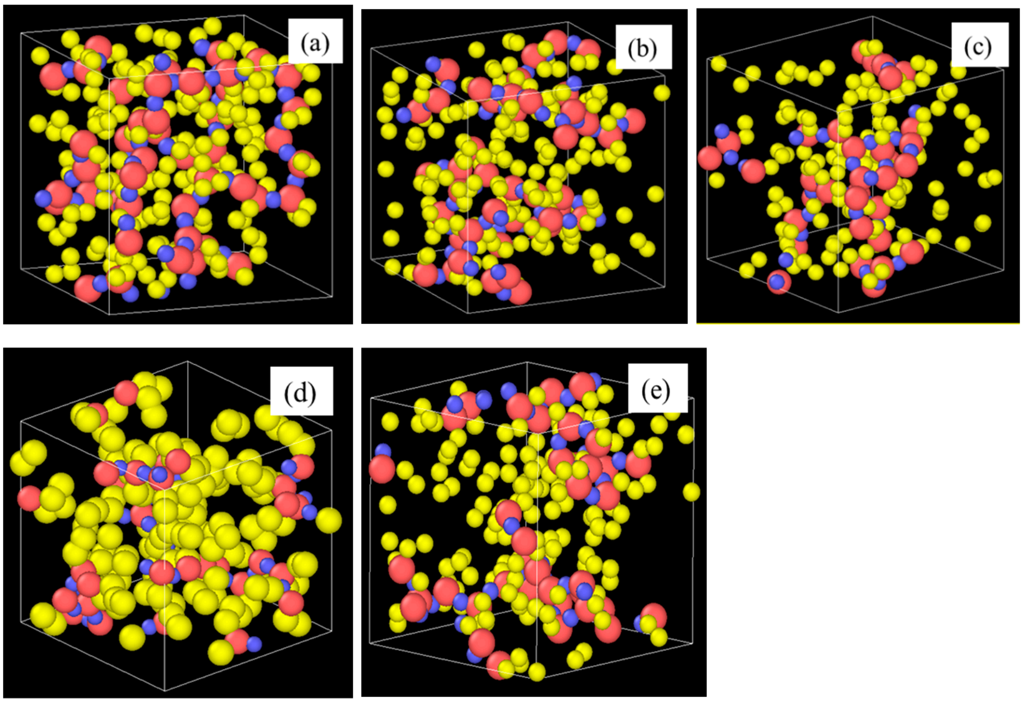

Figure S15. Atomic structural evolution in 3D view for the PDES system at 1500 K simulation temperature: (a) 0 ns, (b) 0.5 ns, (c) 1 ns, (d) 1.5 ns, and (e) 2 ns.

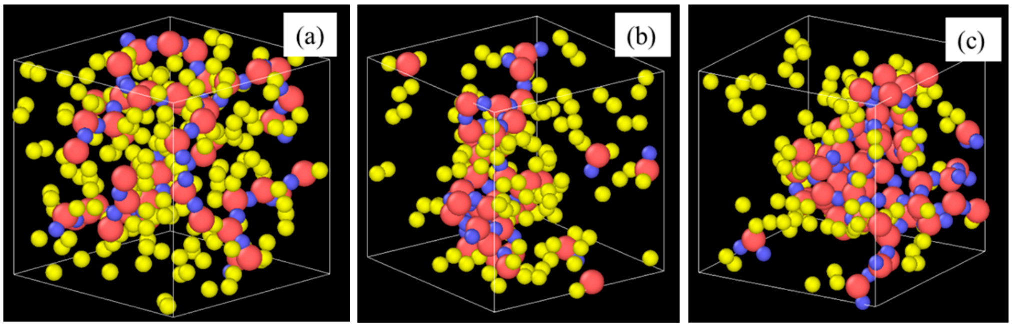

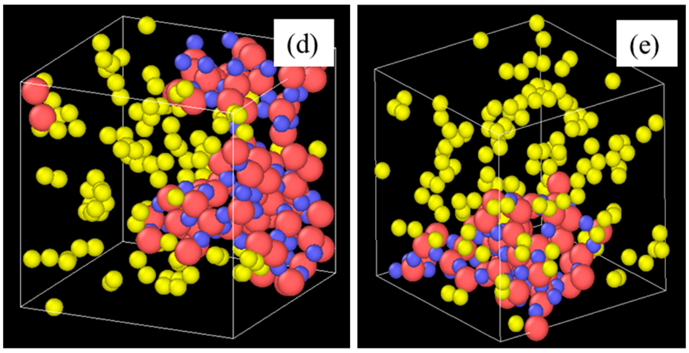

Figure S16. Atomic structural evolution in 3D view for the PDES system at 1800 K simulation temperature: (a) 0 ns, (b) 0.5 ns, (c) 1 ns, (d) 1.5 ns, and (e) 2 ns.

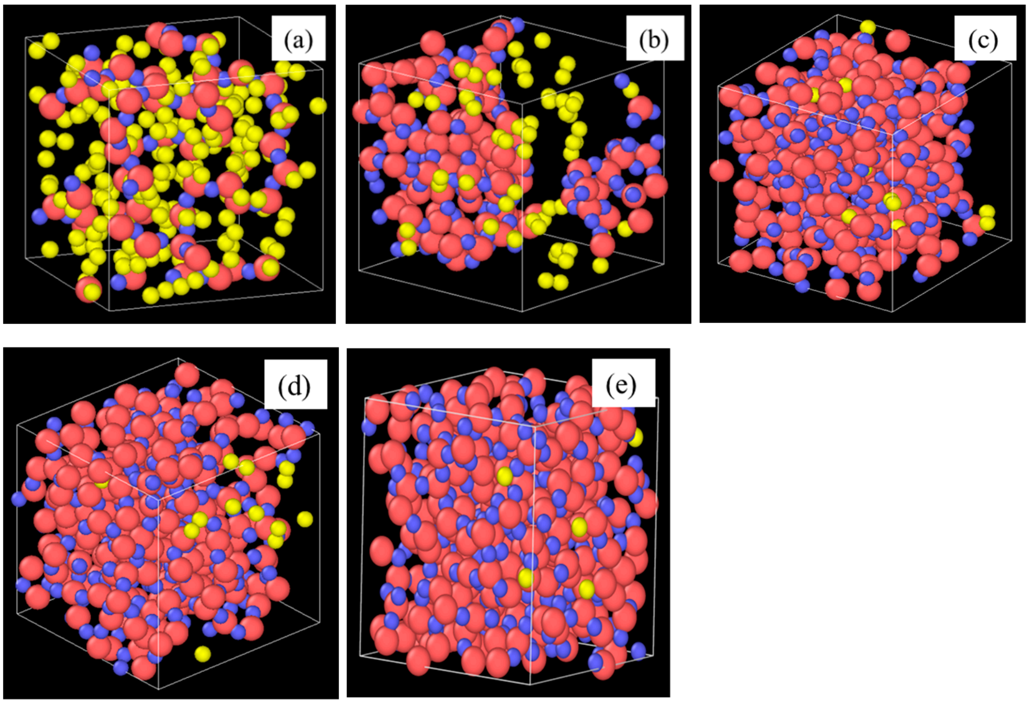

Figure S17. Atomic structural evolution in 3D view for the PDES system at 2100 K simulation temperature: (a) 0 ns, (b) 0.5 ns, (c) 1 ns, (d) 1.5 ns, and (e) 2 ns.

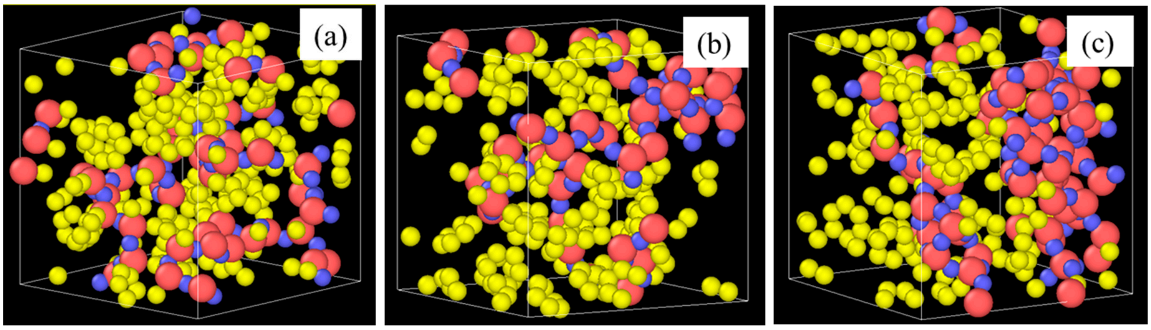

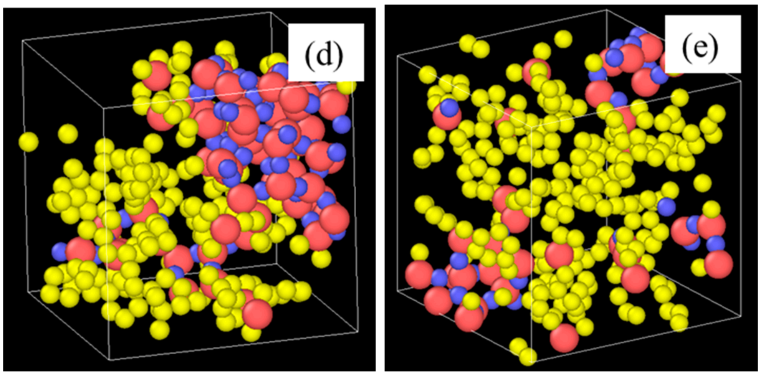

Figure S18. Atomic structural evolution in 3D view for the PMPS system at 1800 K simulation temperature: (a) 0 ns, (b) 0.5 ns, (c) 1 ns, (d) 1.5 ns, and (e) 2 ns.

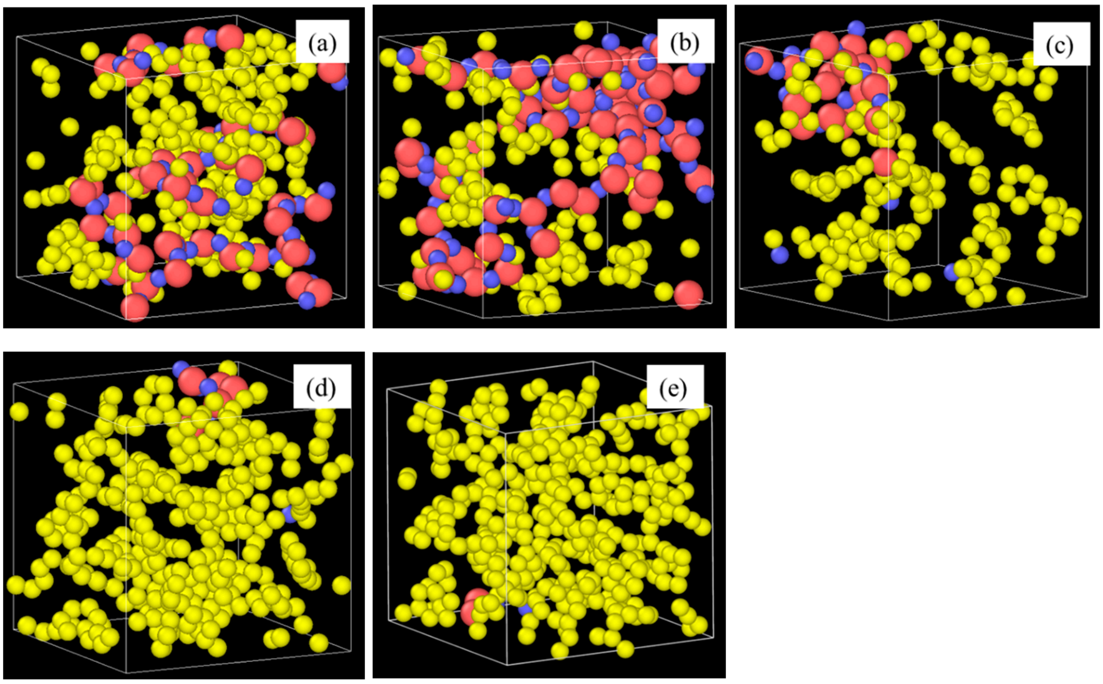

Figure S19. Atomic structural evolution in 3D view for the PMPS system at 2100 K simulation temperature: (a) 0 ns, (b) 0.5 ns, (c) 1 ns, (d) 1.5 ns, and (e) 2 ns.
